# High-efficiency and reliable same-parent thermoelectric modules using Mg_3_Sb_2_-based compounds

**DOI:** 10.1093/nsr/nwad095

**Published:** 2023-04-13

**Authors:** Meng Jiang, Yuntian Fu, Qihao Zhang, Zhongliang Hu, Aibin Huang, Shuling Wang, Lianjun Wang, Wan Jiang

**Affiliations:** State Key Laboratory for Modification of Chemical Fibers and Polymer Materials & College of Materials Science and Engineering, Donghua University, Shanghai 201620, China; State Key Laboratory for Modification of Chemical Fibers and Polymer Materials & College of Materials Science and Engineering, Donghua University, Shanghai 201620, China; Institute for Metallic Materials, Leibniz Institute for Solid State and Materials Research Dresden (IFW Dresden), Dresden 01069, Germany; State Key Laboratory for Modification of Chemical Fibers and Polymer Materials & College of Materials Science and Engineering, Donghua University, Shanghai 201620, China; State Key Laboratory of High Performance Ceramics and Superfine Microstructure, Shanghai Institute of Ceramics, Chinese Academy of Sciences, Shanghai 200050, China; State Key Laboratory for Modification of Chemical Fibers and Polymer Materials & College of Materials Science and Engineering, Donghua University, Shanghai 201620, China; State Key Laboratory for Modification of Chemical Fibers and Polymer Materials & College of Materials Science and Engineering, Donghua University, Shanghai 201620, China; State Key Laboratory for Modification of Chemical Fibers and Polymer Materials & College of Materials Science and Engineering, Donghua University, Shanghai 201620, China; Institute of Functional Materials, Donghua University, Shanghai 201620, China

**Keywords:** Mg_3_Sb_2_, same-parent thermoelectric modules, low-temperature joining and high-temperature service, power generation efficiency, module reliability

## Abstract

Thermoelectric modules can convert waste heat directly into useful electricity, providing a clean and sustainable way to use fossil energy more efficiently. Mg_3_Sb_2_-based alloys have recently attracted considerable interest from the thermoelectric community due to their nontoxic nature, abundance of constituent elements and excellent mechanical and thermoelectric properties. However, robust modules based on Mg_3_Sb_2_ have progressed less rapidly. Here, we develop multiple-pair thermoelectric modules consisting of both n-type and p-type Mg_3_Sb_2_-based alloys. Thermoelectric legs based on the same parent fit into each other in terms of thermomechanical properties, facilitating module fabrication and ensuring low thermal stress. By adopting a suitable diffusion barrier layer and developing a new joining technique, an integrated all-Mg_3_Sb_2_-based module demonstrates a high efficiency of 7.5% at a temperature difference of 380 K, exceeding the state-of-the-art same-parent thermoelectric modules. Moreover, the efficiency remains stable during 150 thermal cycling shocks (∼225 h), demonstrating excellent module reliability.

## Teaser

The stable-service same-parent all-Mg_3_Sb_2_-based thermoelectric modules provide a fresh perspective for the efficient generation of electricity from low-grade but extremely abundant waste heat.

## INTRODUCTION

Thermoelectric (TE) power generation technology based on the Seebeck effect can convert waste heat directly into electricity. It has the advantages of solid-state operation, working without moving parts, free maintenance and extended service. Thus it has been regarded as one of the most promising solutions to improve the utilization efficiency of fossil fuels and alleviate environmental pollution [1,2]. Ideally, the maximum conversion efficiency (*η*_max_) of a TE device depends on the temperature difference across the TE legs and the figure of merit (usually denoted by the capital *Z*) of a TE p–n couple. The latter involves the dimensional factor of the TE legs and TE properties of the constituent materials. Therefore, in order to achieve a high *η*_max_, a large temperature difference, an appropriate geometry and high-performance TE materials are desirable.

Over the past decades, considerable efforts have been made to improve the performance of TE materials, which is gauged by the dimensionless figure of merit *zT* (*zT* = *S*^2^*σT*/*κ*_tot_, where *S, σ, κ*_tot_ and *T* are the Seebeck coefficient, electrical conductivity, total thermal conductivity and absolute temperature, respectively) [3]. In order to obtain higher conversion efficiencies, higher *zT* values are required. To date, a number of excellent TE materials with *zT* > 1.5 have been reported, such as IV–VI compounds (e.g. SnSe, GeTe and PbSe), skutterudites and Cu_2_Se [4–8]. These unprecedented advances in improving the performance of TE materials are an important enabling step that heralds widespread applications of TE devices. However, the advancements in TE device technology have progressed less rapidly. Current efforts are focused primarily on the single-leg or unicouple issues, such as the design of electrodes, screening of barrier layers and interface optimization [2,3]. A single leg is very useful for assessing the potential of a particular TE material, but is still far from practical applications. Towards industrial applications, modules consisting of both n-type and p-type TE materials need to be developed. However, the development of modules is more challenging than the fabrication of a single leg. More issues should be elaborately addressed, such as the development of matching n-type and p-type thermoelectric materials, geometry optimization of TE legs, welding and assembling of multiple legs and evaluation of the efficiency and reliability of modules. In addition, most TE components currently being used or studied contain rare elements (e.g. Te) or toxic elements (e.g. Pb), presenting a potential impediment to large-scale applications.

In recent years, Mg_3_Sb_2_-based compounds have attracted considerable interest from the thermoelectric community due to their nontoxic nature, abundance of constituent elements and excellent mechanical robustness [9]. These compounds were studied as p-type TE materials for a long time until Tamaki *et al.* reported in 2016 that n-type semiconducting behavior was achieved by adding a slight excess of Mg coupled with Te doping [10]. Inspired by this, subsequent research into Mg_3_Sb_2_ has flourished and significant progress has been made over the past 5 years, including the understanding of the origin for excellent power factor and intrinsically low thermal conductivity, the revelation of the carrier scattering mechanism, the increasing awareness of Mg defect chemistry and the improvements in TE performance [9,11]. So far, *zT* values of n-type Mg_3_Sb_2_-based alloys have reached 0.8 at 300 K and exceeded 1.5 at 750 K. In parallel, p-type Mg_3_Sb_2_ has also gained further improvements in *zT*, which approaches 1.0 at 773 K [12]. These cheering results make the low-cost and environmentally friendly Mg_3_Sb_2_-based compounds promising substitutes for the state-of-the-art Te- or Pb-containing alloys towards medium-temperature TE power generation.

The rapid breakthrough in TE properties of Mg_3_Sb_2_-based compounds has recently ignited intensive research interest in their device development. At a single-leg level, efforts have been made in terms of scalable synthesis of n-type Mg_3_(Sb, Bi)_2_, design of reliable junction interfaces and screening of barrier layers [13–15]. A noteworthy result is that a single-leg efficiency of ∼10% could be achieved at a temperature difference of 400 K with a heat source temperature of 700 K [14], indicating good potential for medium-temperature power generation applications. At a unicouple or module level, different p-type TE compounds, such as Bi_2_Te_3_, MgAgSb, GeTe, CdSb and CoSb_3_, have been used for pairing with n-Mg_3_Sb_2_ [16–20]. The modules made from different material combinations have offered outstanding power generation performance in the low- and medium-temperature ranges, opening up new possibilities for efficient waste heat recovery applications. However, it is noticeable that these modules are all fabricated using n- and p-type TE materials based on different parent compounds. Due to the fact that the TE and chemical properties of these n- and p-type TE materials differ significantly, cumbersome device geometry design and individually selecting suitable barrier layers are needed [21,22]. More critically, TE modules for power generation usually operate at large temperature gradients (e.g. 300–500 K for mid-temperature power generation applications [3]) and fluctuating temperatures, so the differences in physical parameters of n- and p-type TE materials, such as the coefficient of thermal expansion (CTE), will result in high thermal stresses that can easily lead to device failure during service [2,3]. In addition, the differences in the melting point and machinability of different n- and p-type TE materials impose additional constraints on the welding and assembly process [3]. Therefore, there is a strong desire to develop efficient and robust TE modules using the same parent TE compounds, so that an excellent match of material properties will facilitate module fabrication and ensure long-term stable operation, and it has been well demonstrated in actual applications, for example, the commercially available Bi_2_Te_3_ modules [1,2], the PbTe modules and SiGe modules used by NASA in deep space exploration [1,3], which are all made from the same parent n- and p-type TE materials.

This motivated the present work to develop novel TE modules consisting of both n-type and p-type Mg_3_Sb_2_-based alloys. Herein, p-type Mg_1.98_Ag_0.02_ZnSb_2_ and n-type Mg_3.2_SbBi_0.996_Se_0.004_ pellets are fabricated by means of mechanical alloying and spark plasma sintering. These two compounds show well-matched thermoelectric and mechanical properties due to their analogous crystal structures and similar chemical compositions. Finite element simulations confirm that the optimum leg cross-sectional area ratio (*A*_p_/*A*_n_) to achieve the maximum conversion efficiency is ∼1.0, which is favorable for the assembly of the modules. Thermomechanical coupling calculations show that the thermal stresses caused by the difference in thermal expansion between the p- and n-type TE elements are minimized. Fe is used as a diffusion barrier layer for both n-type and p-type legs, and a one-step sintering process is adopted to fabricate the TE joints, which enables strong bonding with low interfacial contact resistivity. Moreover, all-Mg_3_Sb_2_-based thermoelectric modules are fabricated by developing a new joining process using Ag composite pastes that allows low-temperature assembly but can withstand higher service temperatures. All these efforts result in a fully Mg_3_Sb_2_-based module with a high efficiency of 7.5% at a heat source temperature of 673 K and exceptional module reliability against thermal cycles.

## RESULTS AND DISCUSSION

### The adaptation of p- and n-type Mg_3_Sb_2_-based compounds

**Figure 1.**
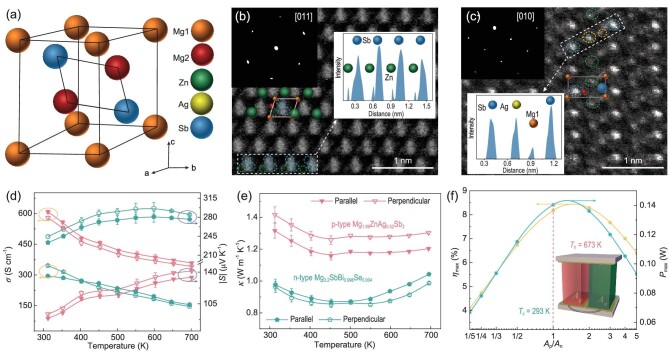
(a) The unit cell diagram of Mg_3_Sb_2_. (b and c) Atomic-resolution STEM images of (b) Mg_2_ZnSb_2_ and (c) Mg_1.98_Ag_0.02_ZnSb_2_ along [011] and [010] orientation, respectively. The insets show Fast Fourier Transform patterns and the linear scanning results based on the detected atomic signal intensity. (d) Electrical conductivity, absolute Seebeck coefficient and (e) thermal conductivity of p-type Mg_1.98_Ag_0.02_ZnSb_2_ and n-type Mg_3.2_SbBi_0.996_Se_0.004_. (f) Simulated maximum conversion efficiency (*η*_max_) and maximum output power (*P*_max_) of a fully Mg_3_Sb_2_-based unicouple as a function of cross-sectional area ratios between p- and n-legs (*A*_p_/*A*_n_). The inset shows the geometrical model for finite element simulation with its boundary conditions. *T*_h_ and *T*_c_ represent the temperature of the hot-side and cold-side ceramic plates, respectively.

To obtain p-type Mg_3_Sb_2_, we first investigated the effect of Zn substitution for Mg (Supplementary Figs S1 and S2), followed by Ag doping (Supplementary Figs S3 and S4). For n-type Mg_3_Sb_2_, the optimization of the TE properties has been detailed in our recent publication [20]. Based on material property manipulation, we further focused on the development of all-Mg_3_Sb_2_-based modules. Here, we chose the compounds with nominal compositions of Mg_1.98_Ag_0.02_ZnSb_2_ (p-type) and Mg_3.2_SbBi_0.996_Se_0.004_ (n-type) to fabricate the modules, which were synthesized by using a scalable routine that combined mechanical alloying and current-assisted sintering (see details in ‘Methods’). The phase composition and microstructure characterization of the samples (Supplementary Figs S5 and S6), including X-ray diffraction, scanning electron microscopy (SEM) and energy dispersive spectroscopy (EDS), indicate good phase purity and uniform elemental distribution. Structural characterization of p-type Mg_1.98_Ag_0.02_ZnSb_2_ at the atomic scale was conducted by using a spherical aberration-corrected transmission electron microscope. The results (Fig. 1a–c) show that Zn and slight Ag, with larger ionic radii than Mg, occupy the Mg2 site of the Mg_3_Sb_2_ crystal lattice. P-type Mg_1.98_Ag_0.02_ZnSb_2_ is confirmed as an inverse *α*-La_2_O_3_-type crystal structure, similar to n-type Mg_3_SbBi [9,23].

Considering that TE modules typically utilize the TE properties of sintered samples along the pressing direction, we prepared large-sized cylinders with a diameter of 10 mm and a height of 12 mm (Supplementary Fig. S7) to perform the TE property measurements. Temperature-dependent electronic and thermal transport properties of p-type Mg_1.98_Ag_0.02_ZnSb_2_ and n-type Mg_3.2_SbBi_0.996_Se_0.004_ are shown in Fig. 1d and e. The n-type material has a lower electrical and thermal conductivity than the p-type, but possesses a higher Seebeck coefficient, resulting in a higher *zT* (Supplementary Fig. S8). In addition, the temperature-dependent transport properties measured along the parallel and perpendicular directions to the pressure are comparable, indicating no significant anisotropy of our Mg_3_Sb_2_ compounds. Furthermore, in terms of module structure, equal cross-sectional areas of the p- and n-type thermoelectric legs (*A*_p_ and *A*_n_), i.e. *A*_p_ = *A*_n_, are preferable. This is because the equal cross-sectional configuration not only facilitates the assembly of the device, but also helps to increase the filling factors of TE legs, thus reducing parasitic heat loss [24]. We note that although the electrical and thermal conductivity values of our p-type and n-type Mg_3_Sb_2_ are different, the simulated maximum conversion efficiency (*η*_max_) and maximum output power (*P*_max_) are both reached at *A*_p_ ≈ *A*_n_ (Fig. 1f). More critically, although other p-type TE materials have higher average *zT* (*zT*_ave_), the corresponding module efficiencies do not reach the maximum for equal cross-sectional configuration (Supplementary Fig. S9). At *A*_p_ = *A*_n_, they even demonstrate lower efficiencies than the full-Mg_3_Sb_2_ combination. Based on the above, we adjust the cross-section area ratio to 1 : 1 for the smooth operation of the device at the cost of losing conversion efficiency and output power slightly. In all, after these material engineering manipulations, our p/n-type Mg_3_Sb_2_ compounds demonstrate excellent adaptations for TE module fabrication.

### Mechanical reliability and bonding interfaces of fully Mg_3_Sb_2_-based modules

In addition to power generation performance, the service reliability of modules is crucial for practical applications. Structural damage, such as cracks in TE legs or interfaces, is often observed during the long-term operation under large temperature differences and mechanical loads, which leads to degraded performance or even structural failure [25]. Such failures are mainly caused by overloaded thermal stresses generated by a mismatch of CTE between different components in the TE module [2,3,26]. To evaluate the mechanical reliability of a fully Mg_3_Sb_2_-based module, we measured the CTE of p-type Mg_1.98_Ag_0.02_ZnSb_2_ and n-type Mg_3.2_SbBi_0.996_Se_0.004_ samples and then carried out finite element simulations on the coupled thermomechanical behavior.

**Figure 2.**
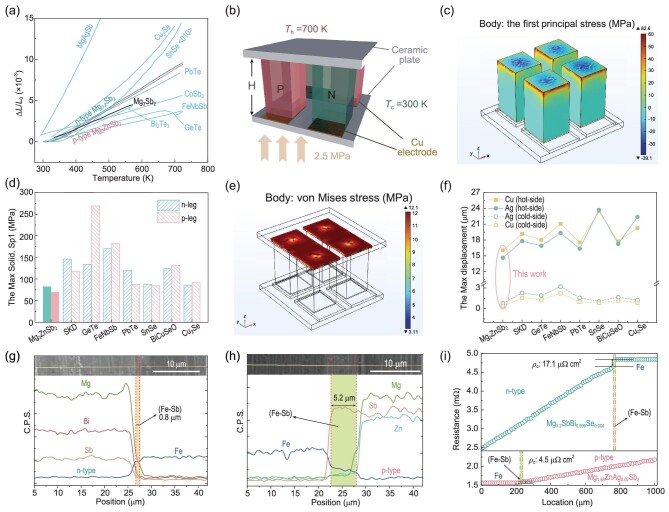
(a) Temperature-dependent relative length variation (Δ*L*/*L*_0_) of p-type Mg_1.98_Ag_0.02_ZnSb_2_ and n-type Mg_3.2_SbBi_0.996_Se_0.004_. Data for some typical thermoelectric compounds are also included for comparisons, such as Bi_2_Te_3_, skutterudites (SKD), GeTe, half-Heusler, PbTe, SnSe, Cu_2_Se, MgAgSb and pristine Mg_3_Sb_2_. (b) Geometrical model and boundary conditions used for the thermomechanical coupling simulations. (c) Distribution of simulated first principal stress in Mg_3_Sb_2_-based materials. (d) Comparison of the maximum first principal stress (Max Solid. Sp1) in TE materials when using n-type Mg_3.2_SbBi_0.996_Se_0.004_ in combination with different p-type TE compounds. (e) Simulated von Mises stress distribution in the electrodes and solders (elastic-plastic materials) of a Mg_3_Sb_2_-based module. (f) Comparison of the maximum displacements in different components when using n-type Mg_3.2_SbBi_0.996_Se_0.004_ in combination with different p-type TE compounds. SEM images and EDS line scanning results of the TE junctions: (g) n-type Mg_3.2_SbBi_0.996_Se_0.004_/Fe and (h) p-type Mg_1.98_Ag_0.02_ZnSb_2_/Fe. (i) Linear scanning of resistance across the junctions for estimating the contact resistivity (*ρ*_c_).

The temperature-dependent relative length change (Δ*L*/*L*_0_) of different TE materials is shown in Fig. 2a and Supplementary Fig. S10, where the average value of the linear thermal expansion coefficient (*α*_L_) can be determined. A higher Δ*L*/*L*_0_ indicates a larger *α*_L_; a closer Δ*L*/*L*_0_ means a better CTE match. It is obvious that our p-type Mg_1.98_Ag_0.02_ZnSb_2_ and n-type Mg_3.2_SbBi_0.996_Se_0.004_ are better matched than the pristine Mg_3_Sb_2_, whose *α*_L,300__–7__00 K_ is measured to be 22.4 × 10^−6^ and 22.7 × 10^−6^ K^−1^, respectively. By contrast, some high-performance p-type TE materials, such as CoSb_3_ and GeTe, show lower *α*_L_ than Mg_3_Sb_2_, in spite of their higher *zT* values. Such CTE mismatch will result in large thermal stress, causing drastic breaks when subjected to frequent thermal cycling. Although thermal stress can be reduced somewhat by optimizing the structural factors such as the geometry of TE legs and the thickness of electrodes [27,28], this will increase the processing difficulty and assembly complexity of the module. In this regard, n- and p-type TE pellets with the same geometry are preferred and cuboidal TE legs are commonly used due to the ease of manufacturing.

To further quantitatively assess the magnitude of thermal stresses, we adopted finite element simulations to calculate the stresses and their distribution within a TE module. A typical module consisting of two n-type and two p-type TE legs is used for the modeling (Fig. 2b and Supplementary Figs S11 and S12) and its geometrical details are shown in Supplementary Table S1. A steady-state temperature gradient is applied to the cold and hot sides of the module with mechanical boundary conditions referring to previous work [29]. The first principal stress (Solid. Sp1) and von Mises stress are used as the failure criteria for estimating the mechanical reliability of TE materials and electrodes/solders, respectively; considering that semiconductor TE materials are brittle in nature with low fracture toughness, electrodes/solders are still ductile metal components [29,30]. In application scenarios, the smaller the thermal stress, the lower the module-broken probability. Large stresses can lead to structural damage, such as cracks at the interface, or to device failure in a worse case, such as detachment of electrodes or breakage of the TE legs [2,3,26].

As shown in Fig. 2c and Supplementary Fig. S13, the maximum Solid. Sp1 occurs at the hot-side outer edges of the TE pellets. The values for n- and p-type legs are 83 and 70 MPa, respectively, within an all-Mg_3_Sb_2_-based module, which is lower than that of the other combinations (Fig. 2d). It is worth noting that although the maximum Solid. Sp1 values are not high when Cu_2_Se and SnSe are used as the p-type TE legs, the issues of phase transition in Cu_2_Se and the anisotropic TE properties of SnSe still block their full development. Figure 2e and Supplementary Fig. S14 show the von Mises stress distribution in the electrodes and solders. The maximum von Mises stress appears in the central region of the contact surfaces between the electrodes and the solders (or TE legs) due to the CTE mismatch between them (Cu ∼16.7 × 10^−6^ K^−1^, Ag ∼19 × 10^−6^ K^−1^ and Mg_3_Sb_2_-based materials ∼22 × 10^−6^ K^−1^). But the maximum stress value of 12 MPa is lower than the previously reported data (e.g. ∼50 MPa between Bi_2_Te_3_/Cu, ∼100 MPa between half-Heusler/Cu) [31,32]. Further, we extracted the displacement along the *x*/*y* direction, eliminating the displacement of the rigid body, to quantify the deformation of these metal components (Fig. 2f and Supplementary Fig. S15). The TE module based on Mg_3_Sb_2_ has a maximum displacement of 15 μm on the hot side and 0.5 μm on its cold side. Compared with other combinations, the same-parent module exhibits superior thermomechanical reliability.

Although the p-type and n-type Mg_3_Sb_2_ materials belong to the same parent, they need doping or substituting with different elements to optimize the thermoelectric properties. These doping or substitution elements do not change the crystal structure and physical properties (e.g. melting point, CTE) of the matrix material, but would have effects on the interfacial diffusion or reaction, leading to differences in interfacial contact resistivity [3,26,33]. Herein, Fe is chosen as the barrier layer material. One-step sintering is used to prepare the junctions for microstructural characterization and contact resistance measurements. As shown in Fig. 2g and h and Supplementary Figs S16–S19, a thin reaction layer is formed between Fe and TE materials after sintering, with thicknesses of 0.8 and 5.2 μm in the n- and p-type TE junctions, respectively. The reaction layers are mainly composed of Fe and Sb elements. The presence of the reaction is beneficial for strong interfacial bonding [34]. Considering the similar composition of the reaction layer in both n- and p-type junctions, the greater thickness in the latter is due to the weaker bonding of the Zn–Sb bonds leading to more Sb being involved in the reaction during sintering [35]. As shown in Fig. 2i, *ρ*_c_ for n- and p-type junctions is 17.1 and 4.5 μΩ·cm^2^, respectively. It is noteworthy that *ρ*_c_ at the Fe/p-type Mg_1.98_Ag_0.02_ZnSb_2_ interface is lower despite the thicker reaction layer. This should be attributed to the different bonding mechanisms between the n- and p-type materials with Fe. The reaction layer between Fe and n-type Mg_3.2_SbBi_0.996_Se_0.004_ is thin and negligible. The work function of Fe is greater than that of Mg_3.2_SbBi_0.996_Se_0.004_, thus resulting in the Schottky potential barrier at the interface [36]. In contrast, a thicker intermediate layer of FeSb_2_ (Supplementary Fig. S18) is produced between the p-type Mg_1.98_Ag_0.02_ZnSb_2_ and Fe. Due to the fact that FeSb_2_ is a p-type semiconductor above room temperature with good electrical conductivity [37], a low contact resistance is thus guaranteed.

Having determined the interface bonding design and the results of contact resistivity, we then use finite element simulation to investigate the effect of Fe on thermal stress and to determine the appropriate height of the TE legs. As shown in Supplementary Fig. S20, the maximum Solid. Sp1 on the thermoelectric legs is increased from 82.6 to 92.5 MPa under the same boundary conditions. The von Mises stresses and the displacements on the electrodes do not change significantly. Overall, the introduction of the Fe layer has little effect on the stress distribution and deformation. Clearly, *η*_max_ and maximum power density (*P*_d, max_) show opposite trends as a function of *H*/*A*_pn_ (Supplementary Fig. S21). This is because the increase in leg height enlarges the effective temperature difference across the TE legs, leading to an increase in *η*_max_. However, the module resistance is also augmented, resulting in a decrease in *P*_d, max_. Finally, considering the module efficiency and the convenience of processing, we adopted the size of 3 mm × 3 mm × 5.5 mm, i.e. *H*/*A*_pn_ = 0.3 mm^−1^, for both n- and p-type TE legs to fabricate the modules.

### Assembly of fully Mg_3_Sb_2_-based modules

**Figure 3.**
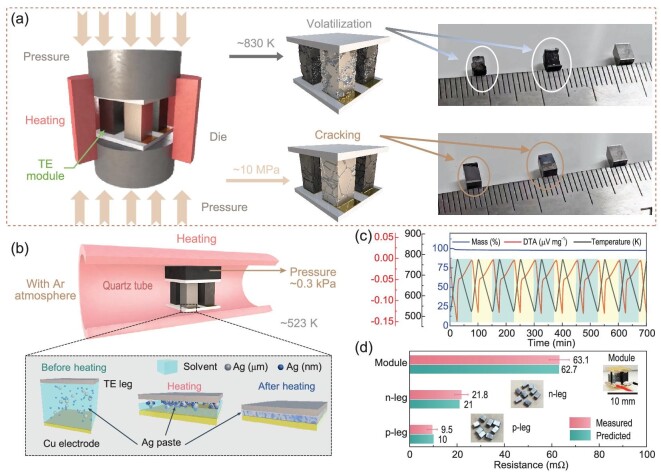
(a) Schematic diagram showing the assembly of a Mg_3_Sb_2_-based module by pressure-assisted high-temperature sintering. Al–Si–Cu brazing fillers are used. (b) Assembly of a Mg_3_Sb_2_-based module by low-temperature and low-pressure joining with the aid of Ag composite paste. (c) Mass change (TG) and heat flow (DSC) for Ag composite paste during 10 thermal cycles from 523 to 773 K. (d) Comparison of the change in resistance before and after soldering with Ag composite paste.

After the preparation of the TE legs, another problem that needs to be solved is how to connect them to the electrodes. Soldering or brazing is by far the most common joining technique. In order to connect Mg_3_Sb_2_ compounds to the electrode, the melting temperature of the brazing filler should be ∼750 K, because melting below this temperature will lead to remelting of the brazing filler and module collapse in service, while melting above this temperature will lead to thermal degradation of the Mg_3_Sb_2_ compounds due to elemental volatilization during the joining process. However, very few candidates exist in the melting range of interest for Mg_3_Sb_2_ according to the solidus and liquidus temperatures in both solder and braze [38]. We found Al–Si–Cu alloys can be used for brazing at ∼830 K and tried to achieve the connections by using pressure-assisted sintering (Fig. 3a), expecting a high-quality joint without raising the joining temperature further. As a result, although the Mg_3_Sb_2_ compounds and Cu electrodes can be connected under 830 K and 10 MPa, severe volatilization of Mg and Zn elements occurred due to the high joining temperature and cracks on the TE legs were observed. We tried to reduce the pressure, but the problem of elemental volatilization remains. We also tried to reduce the joining temperature or time, but the junctions could not be connected.

To address this issue, a further attempt was made to adopt a low-temperature and low-pressure joining technique with the aid of Ag composite paste. The Ag composite paste is composed of nanosized Ag particles, micron-sized Ag particles and organic solvent (Supplementary Fig. S22), where the nanosized Ag particles are used as a ‘binder’ to enhance the joining driving force and the micron-sized Ag particles act as a ‘framework’ for the solder layer [38,39]. As shown in Fig. 3b, Ag paste is uniformly coated on the electrodes before heating and four TE legs are mounted on the surface of the paste. The joining process is carried out in a tube furnace with a joining temperature of 523 K (Supplementary Fig. S23) and a holding time of 1.5 h under Ar atmosphere. To fix the TE module during the joining process, we added a graphite block with a weight of 3.5 g, corresponding to 0.3 kPa, onto the TE module. The pressure caused by the graphite block is much lower than those used in typical pressure-assisted joining processes (e.g. on the order of MPa, Supplementary Table S3). During the heating process, the organics gradually decompose and the surface energy and chemical potential of the nano-Ag particles begin to decrease. The sintering neck is then formed between the closely distributed Ag particles (Supplementary Fig. S24). Finally, the curvature of the sintering neck approaches almost infinity, and the bonding between TE legs and Cu electrodes is gradually completed (Supplementary Fig. S25a and b). In order to confirm that the Ag composite paste can meet the operating temperature of Mg_3_Sb_2_-based modules, we conducted 10 thermal cycling tests from 523 to 773 K on the sintered paste. As shown in Fig. 3c, the composite paste exhibits good thermal stability. In addition, we compare the change in resistance before and after joining with Ag composite paste to ensure that TE materials and interfaces are not damaged. The resistances of as-prepared individual n- and p-type TE legs are measured to be 21.8 and 9.5 mΩ, respectively (Fig. 3d), consistently with the predicted values based on material resistivity. The measured resistance of a fully Mg_3_Sb_2_-based module bonded with Ag composite paste is 63.1 mΩ. This is comparable to the calculated value of 62.7 mΩ, implying that this low-temperature, low-pressure joining technique with the aid of Ag composite paste works well.

Furthermore, though Mg_2_Cu, Fe_7_Mg_2_Cr and Nb have recently been reported to be better barrier layer materials for Mg_3_Sb_2_, demonstrating lower contact resistivity and excellent interface stability, there are still complicated diffusion mechanisms to be further explored [15,20,40]. When the Fe powder forms a dense Fe layer after sintering in argon, the stable contact resistance is guaranteed. The protocol of our Ag composite paste as well as the joining technique is general and feasible for these newly developed conductive barrier layers, as silver and its alloys have proven excellent solderability [41]. An additional advantage of our Ag composite paste is that it can be used for soldering at lower temperatures (∼523 K) and smaller pressures (Supplementary Table S3), ensuring that the TE legs are intact during the soldering process. This approach effectively solves the long-standing problem of not having a suitable solder for mid-temperature (500–800 K) TE modules, which can be extendable to a wider range of electrode/TE material combinations, such as PbTe, SnSe, GeTe or skutterudite systems.

### Module power generation performance and reliability

**Figure 4.**
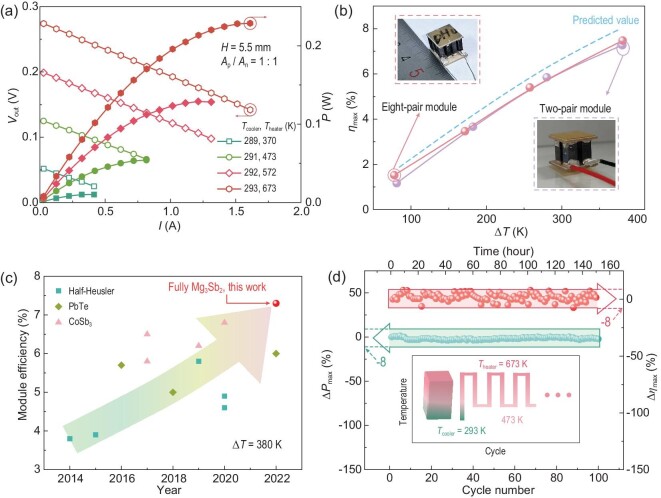
(a) Output voltage (hollow) and output power (solid) of a fully Mg_3_Sb_2_-based module as a function of current under different measuring temperature differences. (b) Measured and predicted conversion efficiency as a function of temperature difference of our two-pair and eight-pair modules. (c) Comparison of the efficiency of existing same-parent TE modules at a temperature difference of 380 K over the last decade. (d) Change of the maximum output power (Δ*P*_max_) and efficiency (Δ*η*_max_) of the fully Mg_3_Sb_2_-based module during 100 thermal cycles when the hot-side temperature (*T*_heater_) cycles between 673 and 473 K and the cold-side temperature (*T*_cooler_) is fixed at 293 K.

The power generation performance of a two-pair module made using all-Mg_3_Sb_2_-based materials was characterized at different temperature gradients, where the heat source temperature (*T*_heater_) was increased from 370 to 673 K and the temperature of the cold-side Cu block (*T*_cooler_) was maintained at ∼290 K. The measured current (*I*)-dependent output voltage (*V*_out_) is shown in Fig. 4a, which exhibits a good linear relationship. For each *I*–*V* curve, the *y*-intercept and the slope represent the open-circuit voltage (*V*_oc_) and the module's internal resistance (*R*_in_) at the assigned temperature difference (Δ*T*), respectively. *P*_max_ is reached when the external electronic load is matched with *R*_in_. As shown in Supplementary Fig. S25c, *V*_oc_ increases from 54 to 276 mV with increasing temperature difference. The measured data are in line with the simulated values obtained from the material properties, suggesting a negligible temperature difference loss between the contacts and good chemical stability of Mg_3_Sb_2_-based materials. *R*_in_ also increases with increasing temperature difference, from 69 to 83 mΩ. This is mainly due to the metallic conductive behavior of our Mg_3_Sb_2_-based materials. The discrepancy between the measured *R*_in_ and predicted *R*_in_ arises from the contact resistances. Supplementary Fig. S26 illustrates the *P*_d,max_ (counting the occupied area of the whole module) and *η*_max_ of the module as a function of temperature difference. *P*_d, max_ increases from 0.01 W cm^−2^ (Δ*T* = 80 K) to 0.23 W cm^−2^ (Δ*T* = 380 K). A high *η*_max_ of 7.3% (Fig. 4b) is obtained at Δ*T* = 380 K, which is higher than that of state-of-the-art TE modules based on the same parent compounds, such as half-Heusler, PbTe and skutterudites, under the same temperature gradient (Fig. 4c and Supplementary Table S4) [24,42–52]. Further, we fabricated an eight-pair all-Mg_3_Sb_2_-based module (Fig. 4b), which achieves a more competitive conversion efficiency of ∼7.5% at the same temperature difference. Moreover, the measured efficiency approaches 90% of the predicted value, indicating a low performance loss thanks to the rational design reaching a higher filling factor of ∼52% and an effective bonding technique.

Furthermore, the long-term reliability of the module is investigated (Fig. 4d and Supplementary Fig. S27). The thermal cycling tests show that both *P*_max_ and *η*_max_ remain stable and comparable to those before thermal cycling. They show fluctuations of ∼8%, which is within the measurement error of the equipment [20]. Compared with previous Mg_3_Sb_2_-based modules (Supplementary Table S5), our reliability results represent a significant improvement. After 100 thermal cycles (∼150 h), we stopped the measurement and disassembled the module to characterize the contact interfaces (Supplementary Fig. S28). As is shown in Supplementary Figs S29 and S30, the Fe/Ag-pastes/Cu interfaces remain intact after thermal cycling and no cracks or significant elemental diffusion are observed, indicating good interfacial stability. Besides, the eight-pair all-Mg_3_Sb_2_-based module could also operate stably and efficiently over 150 thermal cycles, ∼225 h, from 473 to 673 K (Supplementary Fig. S31). Given that this is the first all-Mg_3_Sb_2_-based thermoelectric module to date, the results of thermal cycling are quite encouraging. In addition, it is worth noting that, although high efficiency (7.5%) of this all-Mg_3_Sb_2_-based module is mainly attributed to the n-type compounds, our outcome is readily extended to a wider range of materials and presents greater potential. For example, if we switch to the state-of-the-art n-type Mg_3.17_B_0.03_Sb_1.5_Bi_0.49_Te_0.01_ and p-type (Ca_0.5_Yb_0.25_Ba_0.25_)_0.995_Na_0.005_Mg_2_Bi_1.98_ as the TE legs [53,54], an exceptional *η*_max_ beyond 10% could be obtained (Supplementary Fig. S32).

## CONCLUSION

In summary, TE modules consisting of both n-type and p-type Mg_3_Sb_2_-based alloys are successfully fabricated. They demonstrate a high conversion efficiency of 7.5% at a temperature difference of 380 K and outstanding thermal cycling reliability. This has been enabled by the development of compatible high-performance same-parent TE materials, as well as rational device construction including low thermal stress, highly conductive interfacial contacts and an efficient joining technique. These results successfully illustrate the great potential of developing all-Mg_3_Sb_2_-based modules for the efficient generation of electricity from low-grade but extremely abundant waste heat.

## METHODS

### Material synthesis and module fabrication

High-purity elements of magnesium (pieces, 99.9%, Sinopharm Chemical Reagent Co., Ltd), antimony (powder, 99.999%, Sinopharm Chemical Reagent Co., Ltd), bismuth (powder, 99.999%, Sinopharm Chemical Reagent Co., Ltd), zinc (powder, 99.999%, Sinopharm Chemical Reagent Co., Ltd), silver (powder, 99.99%, Macklin) and selenium (powder, 99%, Sinopharm Chemical Reagent Co., Ltd) were weighed according to the composition of p-type Mg_1.98_ZnAg_0.02_Sb_2_ and n-type Mg_3.2_Sb_1_Bi_0.996_Se_0.004_, and then sealed into stainless-steel ball-milling jars inside a glovebox with argon atmosphere. The mechanical alloying process was undertaken by planetary ball mill equipment (QM-3SP2, Nanjing Nanda Instrument Plant, China) at 480 r/min over 20 h for the synthesis of p-type materials, while oscillating ball mill equipment (MSK-SFM-3-II, Hefei Kejing Material Technology Co., Ltd, China) at 900 r/min over 20 h was used for the synthesis of n-type materials. The ball-milled powders were then added into a cylindrical graphite die with a diameter of 10 mm. Spark plasma sintering (SPS, Dr. Sinter 725, Sumitomo Coal Mining Co., Ltd., Japan) was used to consolidate the powders. The p-type Mg_1.98_ZnAg_0.02_Sb_2_ samples were sintered under a pressure of 50 MPa at 853 K for 5 minutes, and n-type Mg_3.2_Sb_1_Bi_0.996_Se_0.004_ samples were sintered under a pressure of 50 MPa at 1023 K for 3 minutes. The bulk n- and p-type samples with Fe barrier layers were prepared by using the SPS technique following the above sintering parameters. After grinding and polishing, the samples were cut into dices with dimensions of 3.0 mm × 3.0 mm × 5.7 mm (two-pair module) and 1.8 mm × 1.8 mm × 5.7 mm (eight-pair module), with 0.1 mm of Fe on the top and bottom. Both the hot and cold sides of the TE junctions were then soldered with silver paste to double-sided direct-bonded copper alumina plates. The overall size of the module consisting of two couples was 10 mm × 10 mm × 7.7 mm. Boron nitride coating was sprayed onto TE legs prior to the power generation measurements to protect the materials from volatilization. Glass fibers (GXZ aluminosilicate fiber paper) were then filled between TE legs to reduce heat losses due to convection and radiation (Supplementary Fig. S33). Copper wires were soldered to the cold-side Cu electrodes to measure the current and voltage. In addition, the details about characterization and simulation are included in the Supplementary data.

## Supplementary Material

nwad095_Supplemental_File

## References

[1] He J , Tritt TM. Advances in thermoelectric materials research: looking back and moving forward. Science 2017; 357: 1369–78. 10.1126/science.aak9997 28963228

[2] Ohta M , Jood P, Murata M et al. An integrated approach to thermoelectrics: combining phonon dynamics, nanoengineering, novel materials development, module fabrication, and metrology. Adv Energy Mater 2019; 9: 1801304. 10.1002/aenm.201801304

[3] Yan Q , Kanatzidis MG. High-performance thermoelectrics and challenges for practical devices. Nat Mater 2022; 21: 503–13. 10.1038/s41563-021-01109-w 34675376

[4] Su L , Wang D, Wang S et al. High thermoelectric performance realized through manipulating layered phonon-electron decoupling. Science 2022; 375: 1385–9. 10.1126/science.abn8997 35324303

[5] Jiang B , Yu Y, Cui J et al. High-entropy-stabilized chalcogenides with high thermoelectric performance. Science 2021; 371: 830–4. 10.1126/science.abe1292 33602853

[6] Jiang B , Wang W, Liu S et al. High figure-of-merit and power generation in high-entropy GeTe-based thermoelectrics. Science 2022; 377: 208–13. 10.1126/science.abq5815 35857539

[7] Zhao W , Liu Z, Sun Z et al. Superparamagnetic enhancement of thermoelectric performance. Nature 2017; 549: 247–51. 10.1038/nature23667 28905895

[8] Zhang Z , Zhao K, Wei TR et al. Cu_2_Se-based liquid-like thermoelectric materials: looking back and stepping forward. Energy Environ Sci 2020; 13: 3307–29. 10.1039/D0EE02072A

[9] Li A , Fu C, Zhao X et al. High-performance Mg_3_Sb_2-_*_x_*Bi*_x_* thermoelectrics: progress and perspective. Research 2020; 2020: 1934848. 10.34133/2020/1934848 33623901 PMC7877388

[10] Tamaki H , Sato HK, Kanno T. Isotropic conduction network and defect chemistry in Mg_3+_*_δ_*Sb_2_-based layered Zintl compounds with high thermoelectric performance. Adv Mater 2016; 28: 10182–7. 10.1002/adma.201603955 27690372

[11] Imasato K , Wood M, Anand S et al. Understanding the high thermoelectric performance of Mg_3_Sb_2_-Mg_3_Bi_2_ alloys. Adv Energy and Sustain Res 2022; 3: 2100208. 10.1002/aesr.202100208

[12] Ren Z , Shuai J, Mao J et al. Significantly enhanced thermoelectric properties of p-type Mg_3_Sb_2_ via co-doping of Na and Zn. Acta Mater 2018; 143: 265–71. 10.1016/j.actamat.2017.10.015

[13] Xu C , Liang Z, Shang H et al. Scalable synthesis of n-type Mg_3_Sb_2-_*_x_*Bi*_x_* for thermoelectric applications. Mater Today Phys 2021; 17: 100336. 10.1016/j.mtphys.2020.100336

[14] Zhu Q , Song S, Zhu H et al. Realizing high conversion efficiency of Mg_3_Sb_2_-based thermoelectric materials. J Power Sources 2019; 414: 393–400. 10.1016/j.jpowsour.2019.01.022

[15] Wu X , Han Z, Zhu Y et al. A general design strategy for thermoelectric interface materials in n-type Mg_3_Sb_1.5_Bi_0.5_ single leg used in TEGs. Acta Mater 2022; 226: 117616. 10.1016/j.actamat.2021.117616

[16] Mao J , Zhu H, Ding Z et al. High thermoelectric cooling performance of n-type Mg_3_Bi_2_-based materials. Science 2019; 365: 495–8. 10.1126/science.aax7792 31320557

[17] Ying P , Wilkens L, Reith H et al. Robust thermoelectric module based on MgAgSb/Mg_3_(Sb,Bi)_2_ with an 8.5% conversion efficiency and a 72 K maximum cooling. Energy Environ Sci 2022; 15: 2557–66. 10.1039/D2EE00883A

[18] Bu Z , Zhang X, Hu Y et al. An over 10% module efficiency using non-Bi_2_Te_3_ thermoelectric materials for recovering heat of <600 K. Energy Environ Sci 2021; 14: 6506–13. 10.1039/D1EE02253A

[19] Bu Z , Zhang X, Hu Y et al. A record thermoelectric efficiency in tellurium-free modules for low-grade waste heat recovery. Nat Commun 2022; 13: 237. 10.1038/s41467-021-27916-y 35017505 PMC8752736

[20] Fu Y , Zhang Q, Hu Z et al. Mg_3_(Bi,Sb)_2_-based thermoelectric modules for efficient and reliable waste-heat utilization up to 750 K. Energy Environ Sci 2022; 15: 3265–74. 10.1039/D2EE01038K

[21] Pei J , Cai B, Zhuang HL et al. Bi_2_Te_3_-based applied thermoelectric materials: research advances and new challenges. Natl Sci Rev 2020; 7: 1856–8. 10.1093/nsr/nwaa259 34691527 PMC8290941

[22] Liu Z , Gao W, Guo F et al. Challenges for thermoelectric power generation: from a material perspective. MatLab 2022; 1: 20220003. 10.54227/mlab.20220003

[23] Nan P , Li A, Cheng L et al. Visualizing the Mg atoms in Mg_3_Sb_2_ thermoelectrics using advanced iDPC-STEM technique. Mater Today Phys 2021; 21: 100524. 10.1016/j.mtphys.2021.100524

[24] Xing Y , Liu R, Liao J et al. A device-to-material strategy guiding the ‘double-high’ thermoelectric module. Joule 2020; 4: 2475–83. 10.1016/j.joule.2020.08.009

[25] Xing T , Song Q, Qiu P et al. Superior performance and high service stability for GeTe-based thermoelectric compounds. Natl Sci Rev 2019; 6: 944–54. 10.1093/nsr/nwz052 34691955 PMC8291431

[26] Liu W , Bai S. Thermoelectric interface materials: a perspective to the challenge of thermoelectric power generation module. J Materiomics 2019; 5: 321–36. 10.1016/j.jmat.2019.04.004

[27] Kondaguli RS , Malaji PV. Geometry design and performance evaluation of thermoelectric generator. Eur Phys J Spec Top 2022; 231: 1587–97. 10.1140/epjs/s11734-022-00492-y

[28] Khalil A , Elhassnaoui A, Yadir S et al. Performance comparison of TEGs for diverse variable leg geometry with the same leg volume. Energy 2021; 224: 119967. 10.1016/j.energy.2021.119967

[29] Li J , Huang H, Liu R et al. Influence of structural factors on thermal stress in skutterudite-based thermoelectric module. Funct Mater Lett 2021; 14: 2151013. 10.1142/S1793604721510139

[30] Karri NK , Mo C. Reliable thermoelectric module design under opposing requirements from structural and thermoelectric considerations. J Electron Mater 2018; 47: 3127–35. 10.1007/s11664-017-5934-6

[31] Clin T , Turenne S, Vasilevskiy D et al. Numerical simulation of the thermomechanical behavior of extruded bismuth telluride alloy module. J Electron Mater 2009; 38: 994–1001. 10.1007/s11664-009-0756-9

[32] Black D , Schoensee L, Richardson J et al. Power generation from nanostructured half-Heusler thermoelectrics for efficient and robust energy harvesting. ACS Appl Energy Mater 2018; 1: 5986–92. 10.1021/acsaem.8b01042

[33] Zhang Q , Deng K, Wilkens L et al. Micro-thermoelectric devices. Nat Electron 2022; 5: 333–47. 10.1038/s41928-022-00776-0

[34] Chu J , Huang J, Liu R et al. Electrode interface optimization advances conversion efficiency and stability of thermoelectric devices. Nat Commun 2020; 11: 2723. 10.1038/s41467-020-16508-x 32483181 PMC7264234

[35] Sun Y , Yin L, Zhang Z et al. Low contact resistivity and excellent thermal stability of p-type YbMg_0.8_Zn_1.2_Sb_2_/Fe-Sb junction for thermoelectric applications. Acta Mater 2022; 235: 118066. 10.1016/j.actamat.2022.118066

[36] Yin L , Chen C, Zhang F et al. Reliable n-type Mg_3.2_Sb_1.5_Bi_0.49_Te_0.01_/304 stainless steel junction for thermoelectric applications. Acta Mater 2020; 198: 25–34. 10.1016/j.actamat.2020.07.058

[37] Koirala M , Zhao H, Pokharel M et al. Thermoelectric property enhancement by Cu nanoparticles in nanostructured FeSb_2_. Appl Phys Lett 2013; 102: 213111. 10.1063/1.4808094

[38] Yang F , Hu B, Peng Y et al. Ag microflake-reinforced nano-Ag paste with high mechanical reliability for high-temperature applications. J Mater Sci: Mater Electron 2019; 30: 5526–35. 10.1007/s10854-019-00846-8

[39] Park BG , Jung KH, Jung SB. Fabrication of the hybrid Ag paste combined by Ag nanoparticle and micro Ag flake and its flexibility. J Alloys Compd 2017; 699: 1186–91. 10.1016/j.jallcom.2016.12.295

[40] Yang J , Li G, Zhu H et al. Next-generation thermoelectric cooling modules based on high-performance Mg_3_(Bi,Sb)_2_ material. Joule 2022; 6: 193–204. 10.1016/j.joule.2021.11.008

[41] Way M , Willingham J, Goodall R. Brazing filler metals. Int Mater Rev 2020; 65: 257–85. 10.1080/09506608.2019.1613311

[42] Bartholomé K , Balke B, Zuckermann D et al. Thermoelectric modules based on half-Heusler materials produced in large quantities. J Electron Mater 2014; 43: 1775–81. 10.1007/s11664-013-2863-x

[43] Fu C , Bai S, Liu Y et al. Realizing high figure of merit in heavy-band p-type half-Heusler thermoelectric materials. Nat Commun 2015; 6: 8144. 10.1038/ncomms9144 26330371 PMC4569725

[44] Yu J , Xing Y, Hu C et al. Half-Heusler thermoelectric module with high conversion efficiency and high power density. Adv Energy Mater 2020; 10: 2000888. 10.1002/aenm.202000888

[45] Xing Y , Liu R, Liao J et al. High-efficiency half-Heusler thermoelectric modules enabled by self-propagating synthesis and topologic structure optimization. Energy Environ Sci 2019; 12: 3390–9. 10.1039/C9EE02228G

[46] Jood P , Ohta M, Yamamoto A et al. Excessively doped PbTe with Ge-induced nanostructures enables high-efficiency thermoelectric modules. Joule 2018; 2: 1339–55. 10.1016/j.joule.2018.04.025

[47] Hu X , Jood P, Ohta M et al. Power generation from nanostructured PbTe-based thermoelectrics: comprehensive development from materials to modules. Energy Environ Sci 2016; 9: 517–29. 10.1039/C5EE02979A

[48] Jia B , Huang Y, Wang Y et al. Realizing high thermoelectric performance in non-nanostructured n-type PbTe. Energy Environ Sci 2022; 15: 1920–9. 10.1039/D1EE03883D

[49] Zong P , Hanus R, Dylla M et al. Skutterudite with graphene-modified grain-boundary complexion enhances zT enabling high-efficiency thermoelectric device. Energy Environ Sci 2017; 10: 183–91. 10.1039/C6EE02467J

[50] Zhang Q , Zhou Z, Dylla M et al. Realizing high-performance thermoelectric power generation through grain boundary engineering of skutterudite-based nanocomposites. Nano Energy 2017; 41: 501–10. 10.1016/j.nanoen.2017.10.003

[51] Nie G , Li W, Guo J et al. High performance thermoelectric module through isotype bulk heterojunction engineering of skutterudite materials. Nano Energy 2019; 66: 104193. 10.1016/j.nanoen.2019.104193

[52] Chu J , Huang J, Liu R et al. Electrode interface optimization advances conversion efficiency and stability of thermoelectric devices. Nat Commun 2020; 11: 2723. 10.1038/s41467-020-16508-x 32483181 PMC7264234

[53] Chen X , Zhu J, Qin D et al. Excellent thermoelectric performance of boron-doped n-type Mg_3_Sb_2_-based materials via the manipulation of grain boundary scattering and control of Mg content. Sci China Mater 2021; 64: 1761–9. 10.1007/s40843-020-1559-4

[54] Guo M , Zhai W, Li J et al. High thermoelectric performance of CaMg_2_Bi_2_ enabled by dynamic doping and orbital alignment. Adv Funct Mater 2022; 32: 2200407. 10.1002/adfm.202200407

