# Supplementary material for: High-efficiency and reliable same-parent thermoelectric modules using Mg3Sb2-based compounds
Source: Natl Sci Rev. 2023 Apr 13;10(6):nwad095. doi: 10.1093/nsr/nwad095 (PMC10174719; doi:10.1093/nsr/nwad095)
Supplement: nwad095_Supplemental_File [file nwad095_supplemental_file.pdf]

Supplementary data

High-efficiency and Reliable Same-parent Thermoelectric Modules using Mg_3Sb_2-based compounds

Meng Jiang, Yuntian Fu, Qihao Zhang, Zhongliang Hu, Aibin Huang, Shuling Wang, Lianjun Wang*, and Wan Jiang**

M. Jiang, Y. T. Fu, Z. L. Hu, S. L. Wang, Prof. L. J. Wang, Prof. W. Jiang
State Key Laboratory for Modification of Chemical Fibers and Polymer, Materials & College of Materials Science and Engineering, Donghua University, Shanghai, 201620, China.
E-mail: wanglj@dhu.edu.cn, Tel: 021-67792835
E-mail: wanjiang@dhu.edu.cn, Tel: 021-67792198

Prof. W. Jiang
Institute of Functional Materials, Donghua University, Shanghai 201620, China.

Dr. Q. H. Zhang
Institute for Metallic Materials, Leibniz Institute for Solid State and Materials Research Dresden (IFW Dresden), Dresden, 01069, Germany.
E-mail: q.zhang@ifw-dresden.de, Tel: +4915753849559

Dr. A. B. Huang
State Key Laboratory of High Performance Ceramics and Superfine Microstructure, Shanghai Institute of Ceramics, Chinese Academy of Sciences, Shanghai 200050, China

This supplement contains

Supplementary methods

Supplementary Figures **1-33**

Supplementary Tables **1-6**

Supplementary References

SUPPLEMENTARY METHODS

Characterization: The phase composition and crystal structure were analyzed by X-ray diffraction (XRD, Rigaku D/Max-2550 PC, Japan) with Cu Kα radiation at 40 kV and 100 mA. The densities of consolidated samples were obtained using the Archimedes method with alcohol as the immersion medium. Microstructural observations and elements analyses were conducted using a thermal field emission scanning electron microscope (FE-SEM: MAIA3, Czech Republic). Structural and elemental characterization of bulk samples at the atomic scale were conducted by spherical aberration corrected Transmission Electron Microscope (HITACHI: HF5000, Japan). The temperature dependence of relative length variation ($\Delta L/L_0$) for p- and n-type compounds was measured by a thermal dilatometer (DIL 402C, Netzsch, Germany). The electrical resistivity and Seebeck coefficient were simultaneously measured using ZEM-3 equipment (ULVAC-RIKO, Japan) under a helium atmosphere. The uncertainty of the electrical resistivity and Seebeck coefficient measurements is around 7%. The thermal conductivity (κ) was calculated by $\kappa = DC_p\rho$, where D is the thermal diffusivity measured by a laser flash apparatus (LFA 457, Netzsch, Germany), C_p is the specific heat capacity calculated by the Maier-Kelly polynomial expression that $C_p = \frac{3NR}{M\mathrm{w}}\left(1+1.3\times10^{-4}T-4\times10^{3}T^{-2}\right)$. The uncertainty of κ is estimated to be within 5%, considering the uncertainties for D, C_p and ρ. The combined uncertainties enable an uncertainty of less than 12% for zT. Thermogravimetry-differential scanning calorimetry (TG-DSC) was tested by Netzsch STA 449F3. The contact resistivity was obtained by a linear resistance scanning measurement using a homemade four-probe measurement system. The thermoelectric conversion efficiency and thermal cycling tests of the module were measured using a commercial measurement system developed by Shanghai Fuyue Vacuum Technology Ltd as detailed in our previous work[1]. The efficiency uncertainty is approximately 10% based on our previous analysis[2]. In addition, before being put into use, our measurement system was calibrated by comparison with a commercial Mini-PEM apparatus (Ulvac-Riko, Japan). The comparable measurement results of both instruments on one TE are reported in details in our previous paper[1]. The measurements in this work were carried out in a chamber

filled with Ar at an initial pressure of 200 Pa.

Finite element simulation: The optimal geometry and thermal stress of the TE modules were implemented using 3D finite element analyses through COMSOL Multiphysics software. For the thermal-mechanical simulation, the elastoplastic model for the steady-state thermal stress analysis is used. Temperature-dependent Seebeck coefficient, electrical conductivity, and thermal conductivity for both n- and p-type TE materials are considered. The properties of other materials used for the simulations are shown in **Table S6** below. The meshes were obtained through the software itself. The meshing method was set as physics-controlled mesh and the element size was set as normal, which is mostly used for stress calculation in the literature[3-7].

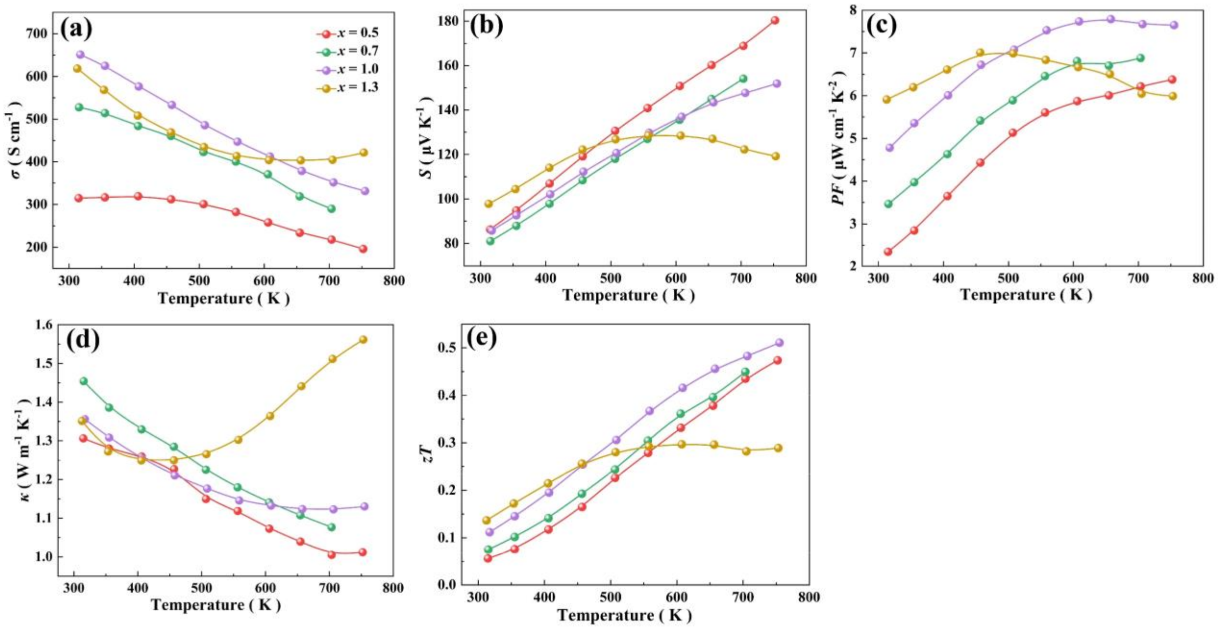

Figure S1. Temperature-dependent **(a)** electrical conductivity, **(b)** absolute *Seebeck* coefficient, **(c)** *PF*, **(d)** κ and **(e)** zT of p-type $Mg_{2.99-x}Na_{0.01}Zn_xSb_2$.

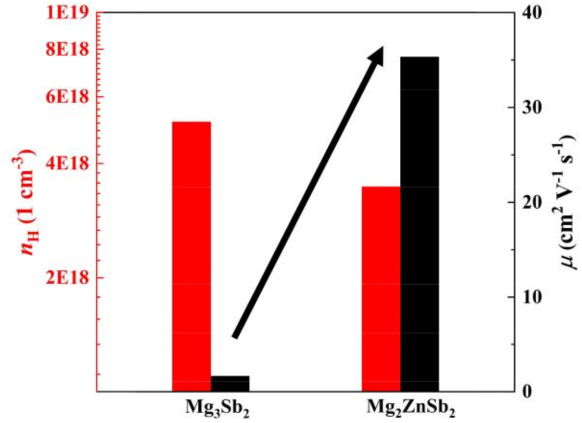

Figure S2. Carrier concentration and mobilities of Mg_3Sb_2 and Mg_2ZnSb_2.

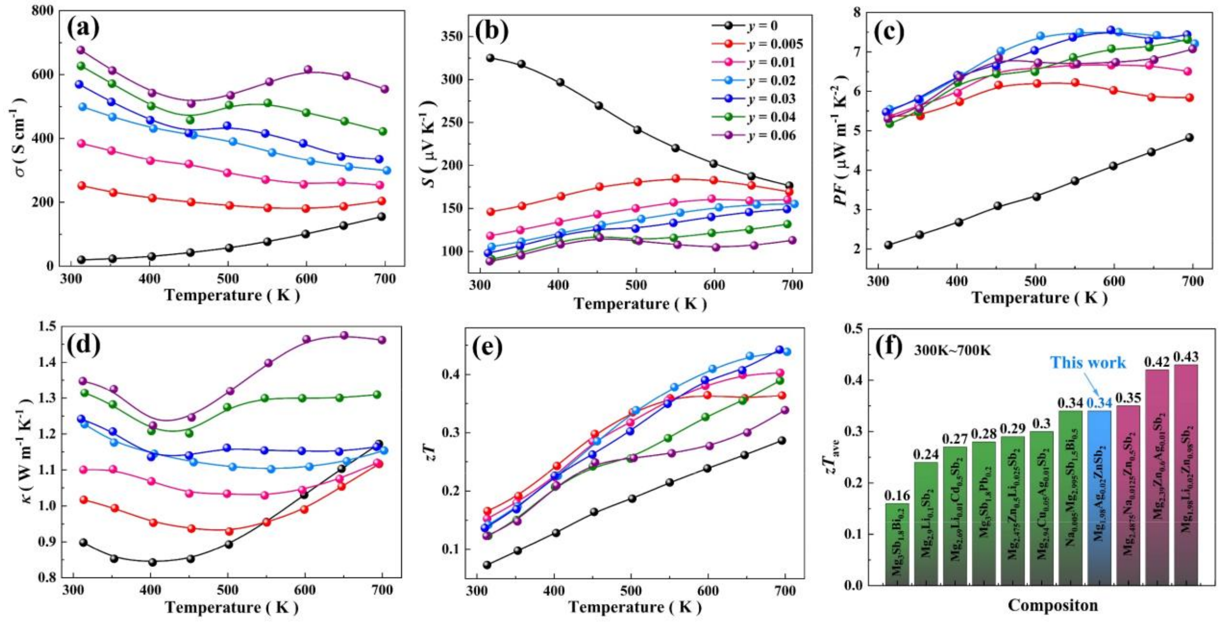

Figure S3. Temperature-dependent **(a)** electrical conductivity, **(b)** absolute *Seebeck* coefficient, **(c)** *PF*, **(d)** κ and **(e)** zT of p-type $Mg_{2-y}Ag_yZnSb_2$. **(f)** comparison zT_{ave} of p-type Mg_3Sb_2 compounds[8-17].

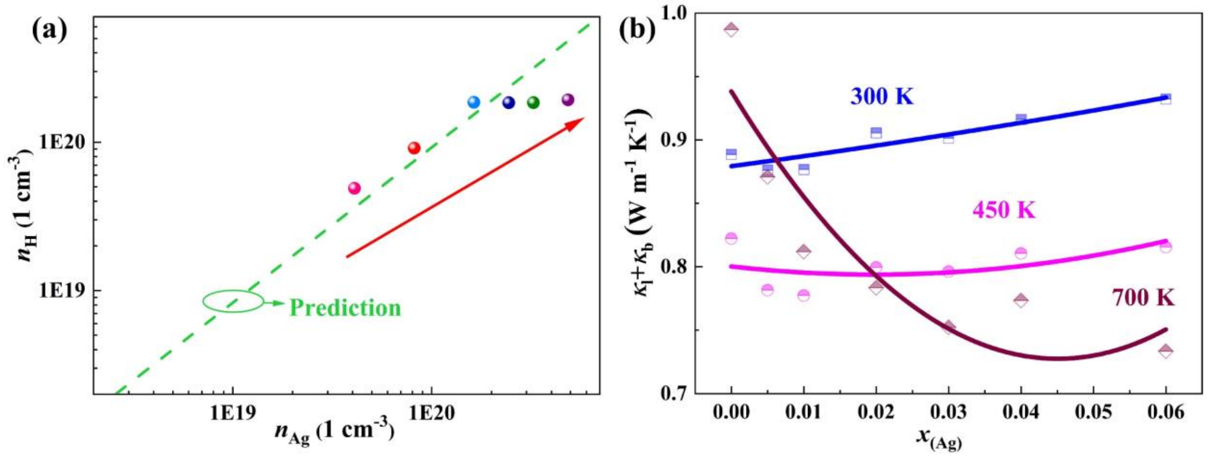

Figure S4. (a) Ag concentration versus expected n_H at room temperature, the green dash line represents the prediction of expected n_H assuming each Ag introduce one hole. **(b)** Composition for Ag of Mg_2ZnSb_2 dependent $\kappa_l+\kappa_b$ at different temperatures, indicating that with the increase of Ag content, the κ_l decreases at 450K and the κ_b decreases at 700K.

The Zn atoms firstly are introduced to replace the Mg2 atoms to weaken the covalent bond polarity of Mg2-Sb, optimizing the carrier mobility **(Figure 1b & S2)**. And the Ag dopants help to promote the carrier concentration, lowering the lattice and bipolar diffusion thermal conductivity **(Figure S3 & S4).** Although some literature reports higher zT_{ave}, it should be noted that these high-zT_{ave} materials are composed of elements such as Pb, Cd, Na, or Li[10-11, 15, 17]. Their widespread use is limited by either the toxicity or high activity of their constituent elements. It is challenging to synthesize these high-performance materials in batch quantities to meet the consumption requirements for module fabrication. A similar situation is encountered in the classical Bi_2Te_3 systems. For example, the zT values reported in the literature for Bi_2Te_3 have exceeded 1.5, whereas Bi_2Te_3 materials currently used in commercial devices have zT of only 1.0. Therefore, from a practical point of view, it is crucial to use materials that are non-toxic, stable, repeatable, and re-producible.

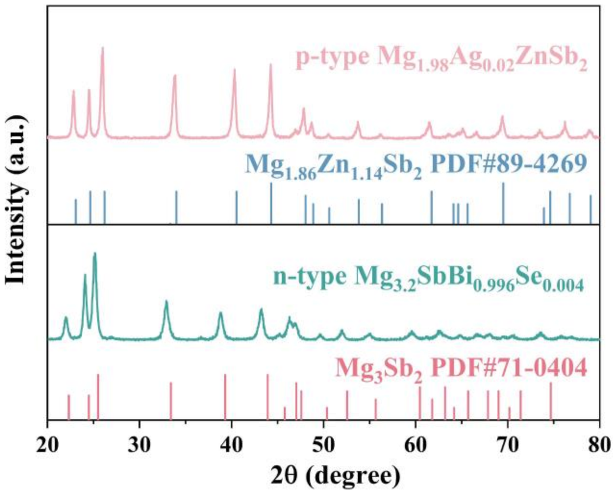

Figure S5. The XRD patterns of bulk n-type $Mg_{3.2}SbBi_{0.996}Se_{0.004}$ and p-type $Mg_{1.98}Ag_{0.02}ZnSb_2$.

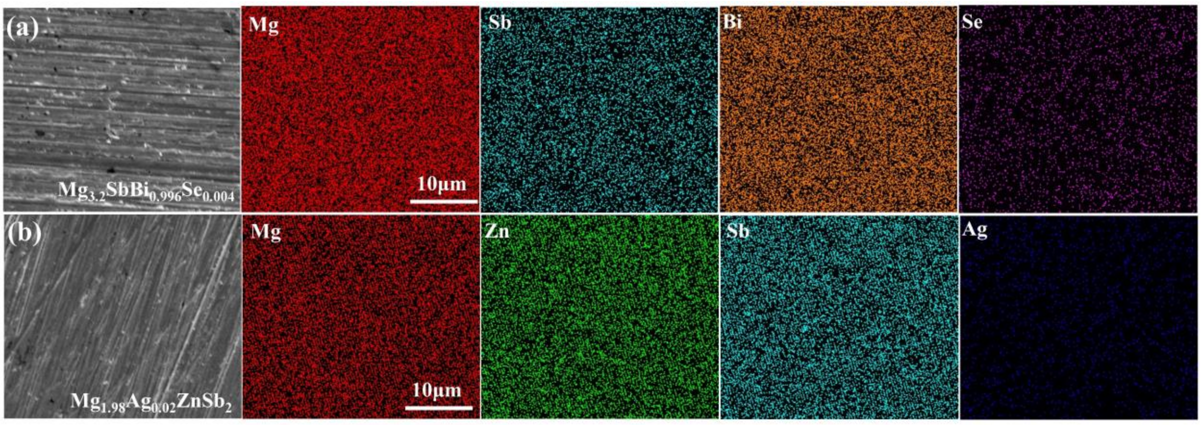

Figure S6. SEM images and the elemental mapping results of the polished surface of bulk samples. **(a)** n-type $Mg_{3.2}SbBi_{0.996}Se_{0.004}$ and **(b)** p-type $Mg_{1.98}Ag_{0.02}ZnSb_2$.

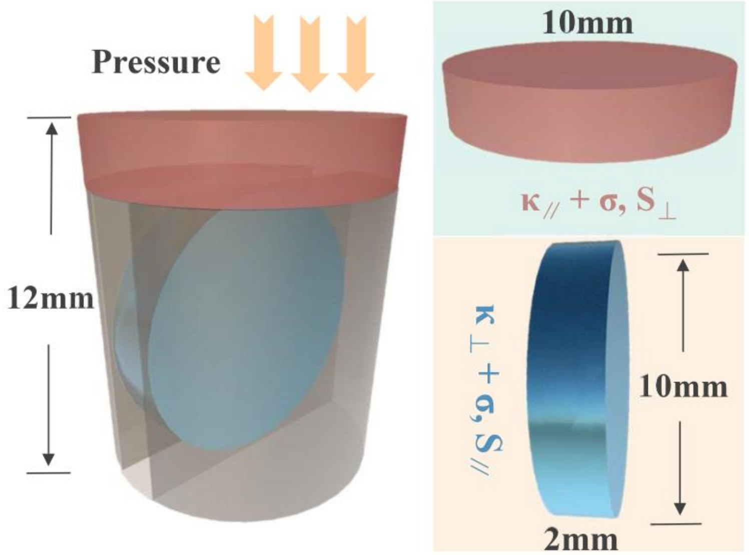

Figure S7. Schematic diagram of large-sized cylinders for the thermoelectric property measurements.

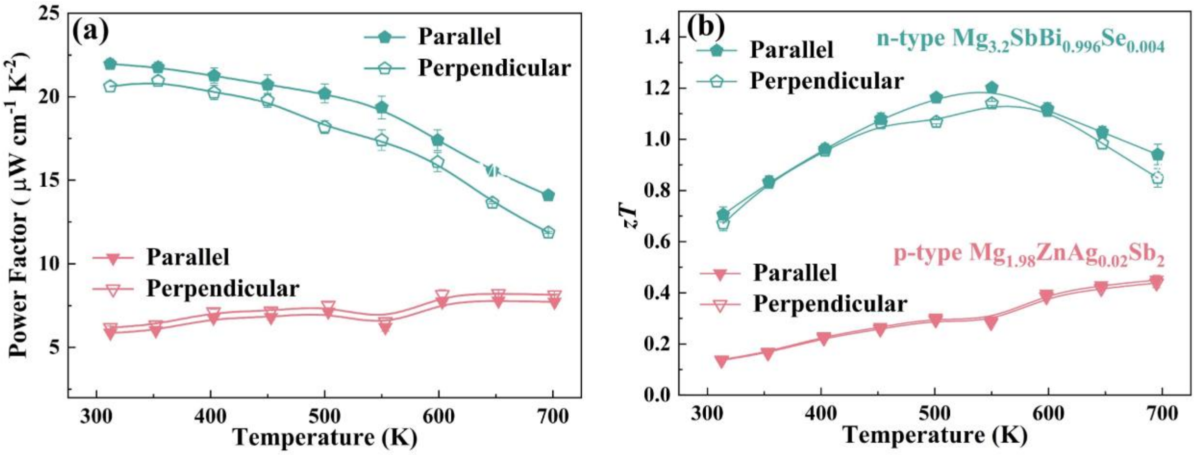

Figure S8. Temperature-dependent transport properties parallel (the solid one) or perpendicular (the hollow one) to the pressure direction. (**a**) power factor; (**b**) zT for n-type $Mg_{3.2}SbBi_{0.996}Se_{0.004}$ and p-type $Mg_{1.98}Ag_{0.02}ZnSb_2$.

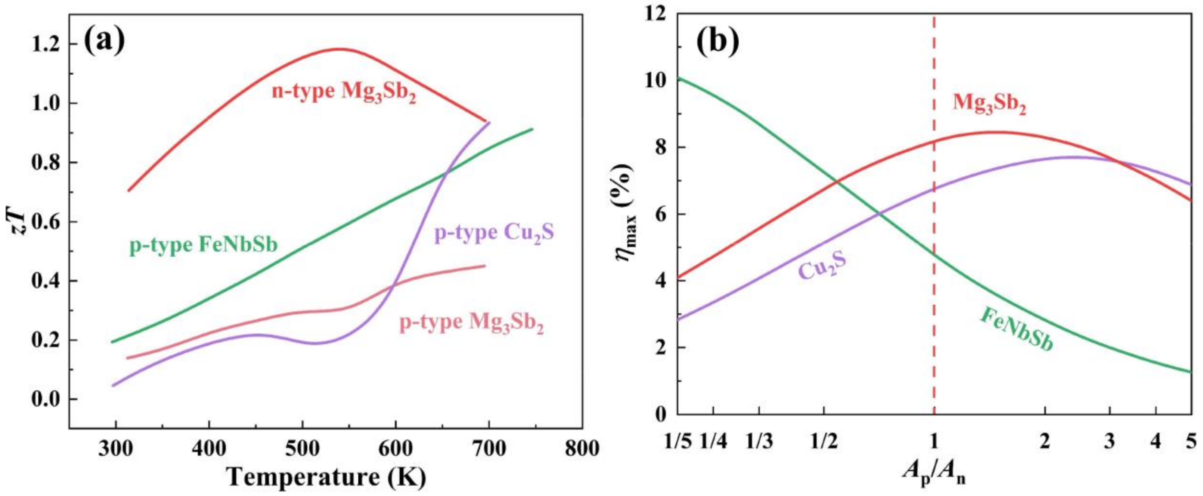

Figure S9. (a) Temperature-dependent *zT*. **(b)** Simulated maximum efficiency of a unicouple based on n-type $Mg_{3.2}SbBi_{0.996}Se_{0.004}$ and different p-type materials ($Mg_{1.98}Ag_{0.02}ZnSb_2$, FeNbSb, Cu_2S) as a function of A_p/A_n[18-19].

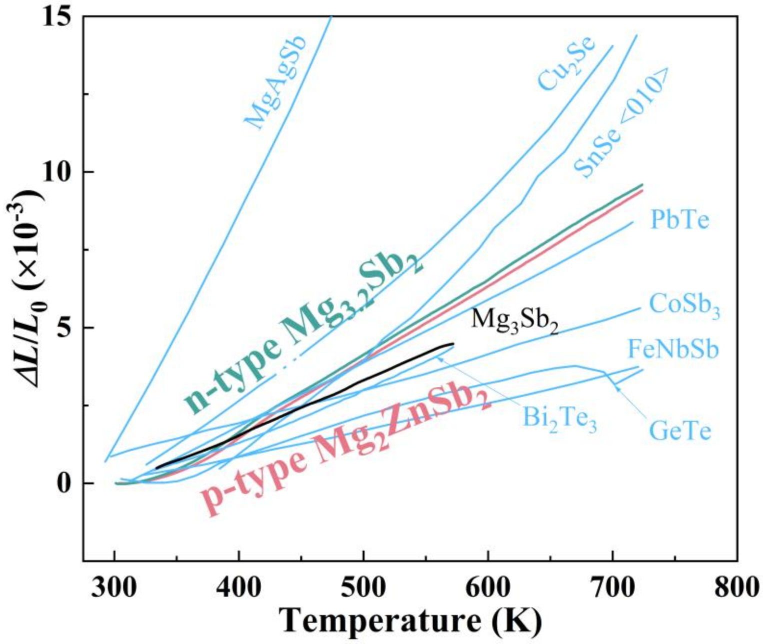

Figure S10. Temperature-dependent relative length variation ($\Delta L/L_0$) of some typical p-type thermoelectric compounds and pristine Mg_3Sb_2[20-29].

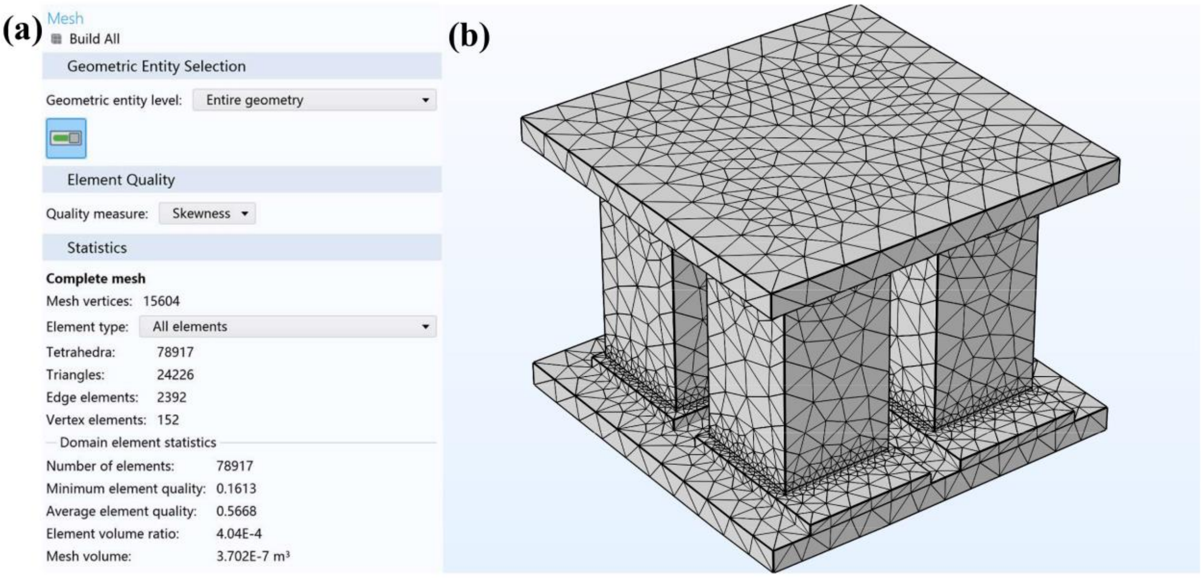

Figure S11. (a) Statistics of the mesh. **(b)** Our model after physics-controlled meshing.

According to the instruction of COMSOL, the *skewness* measure can be used to evaluate the mesh quality, which is a suitable metric for most types of meshes (*https://www.comsol.com/blogs/how-to-inspect-your-mesh-in-comsol-multiphysics?setlang=1*). Generally, a quality of 1 is the best possible and it indicates an optimal element in the chosen quality measure, while 0 represents a degenerated element. As shown in **Figure S11,** the average element quality of our mesh is 0.5668, indicating a good meshing quality. The minimum element quality is 0.1613, which is also greater than the criterion (0.1) for poor meshing in 3D models.

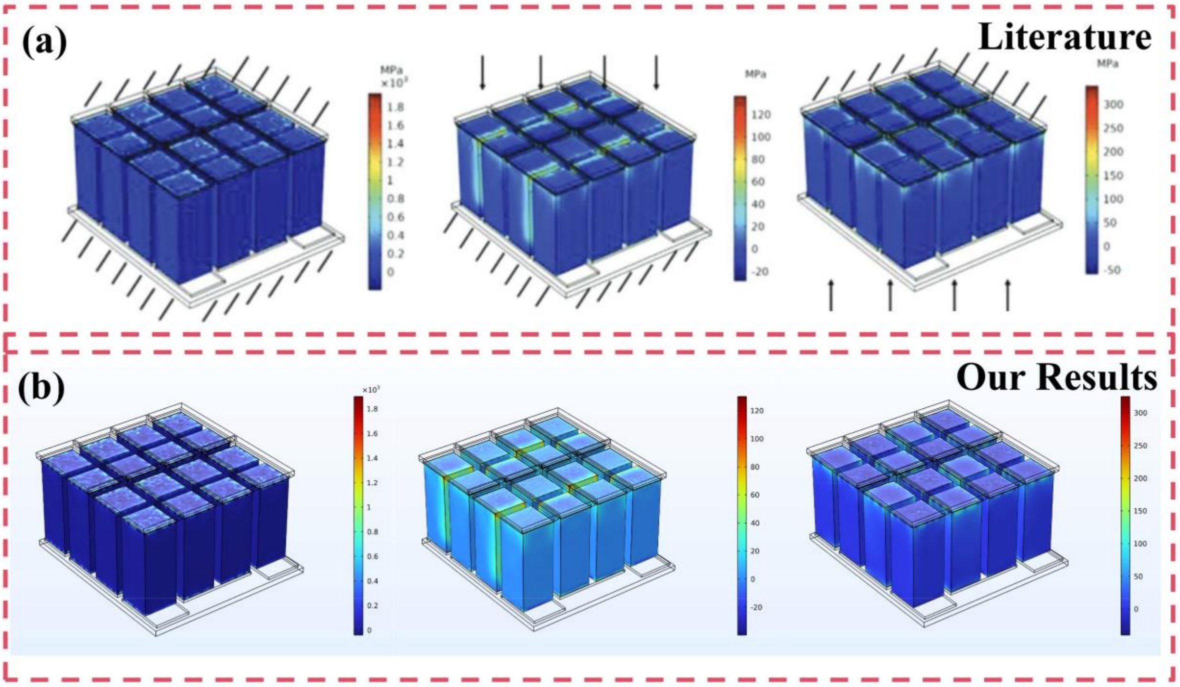

Figure S12. Stress distribution results from **(a)** literature, and **(b)** our model. The boundary conditions from left to right are “both constrained”; “2 MPa on hot side and constrained on cold side”; and “Constrained on hot side and 2 MPa on cold side”, which are the same as those used in the literature[30].

And we have validated our model by constructing the same geometry, using the same material properties and boundary conditions as provided. As shown in **Figure S12 and Table S2**, the profiles of stress distribution are generally the same, and our calculated values agree with the literature in orders of magnitude, confirming the reliability of our model.

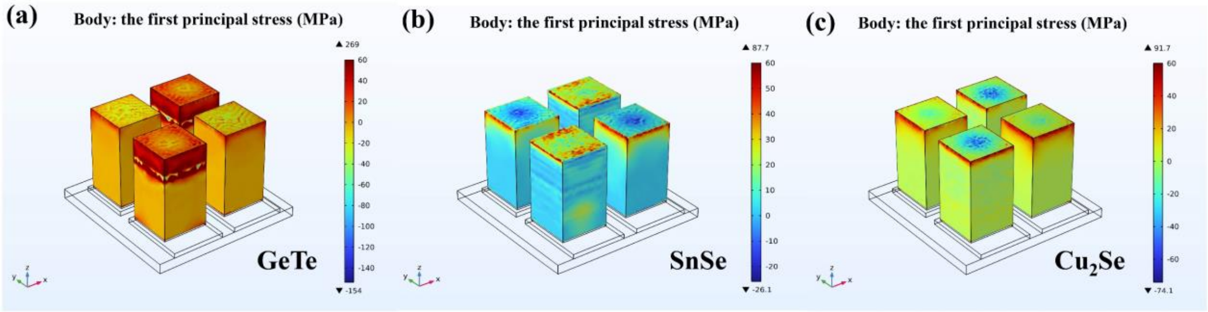

Figure S13. Distribution of simulated first principal stress in TE legs consisting of n-type $Mg_{3.2}SbBi_{0.996}Se_{0.004}$ and different p-type compounds: (**a**) p-type GeTe, (**b**) p-type SnSe, (**c**) p-type Cu_2Se. Material properties used for simulation are shown in **Table S6**.

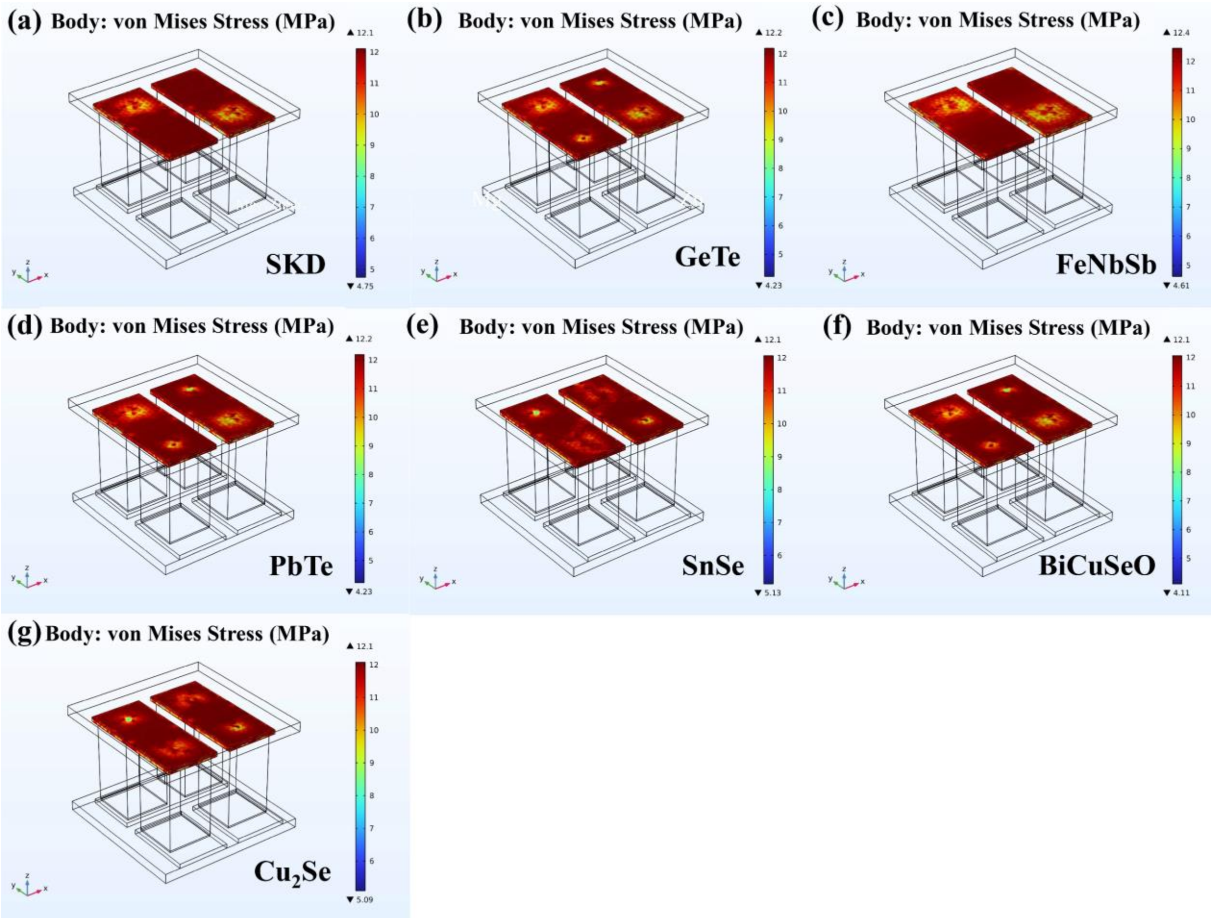

Figure S14. Von Mises stress distribution in the electrodes and solders when using n-type $Mg_{3.2}SbBi_{0.996}Se_{0.004}$ in combination with different p-type TE compounds: **a)** skutterudites, **b)**

GeTe, **c)** half-Heusler, **d)** PbTe, **e)** SnSe, **f)** BiCuSeO,[31] **g)** Cu_2Se. Only the stress distribution at the hot side is shown here as it is more susceptible to damage.

In addition, smaller areas of red-colour concentration in the stress distribution imply less deformation, which ensures a more stable bonding when operating at large temperature gradients.

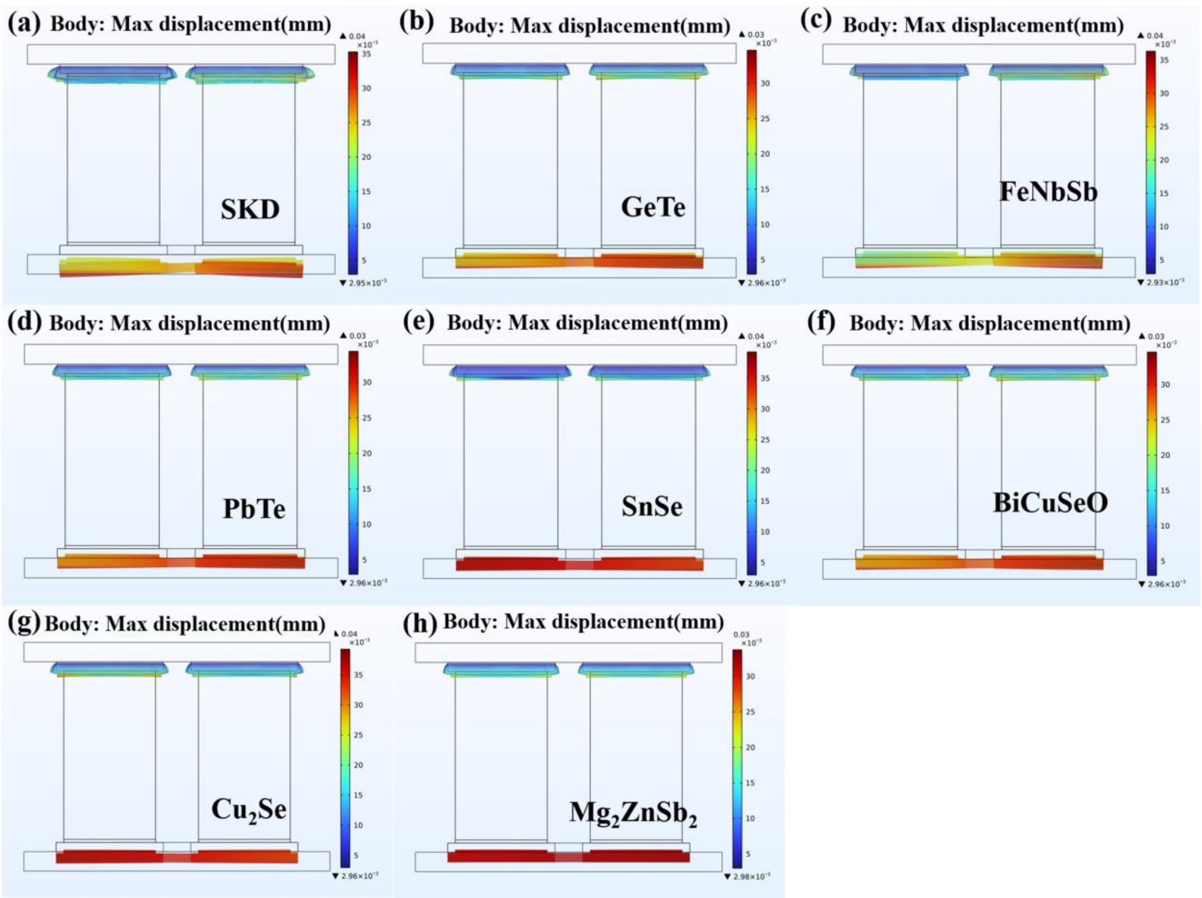

Figure S15. The maximum displacement in different components when using n-type $Mg_{3.2}SbBi_{0.996}Se_{0.004}$ in combination with different p-type TE compounds.

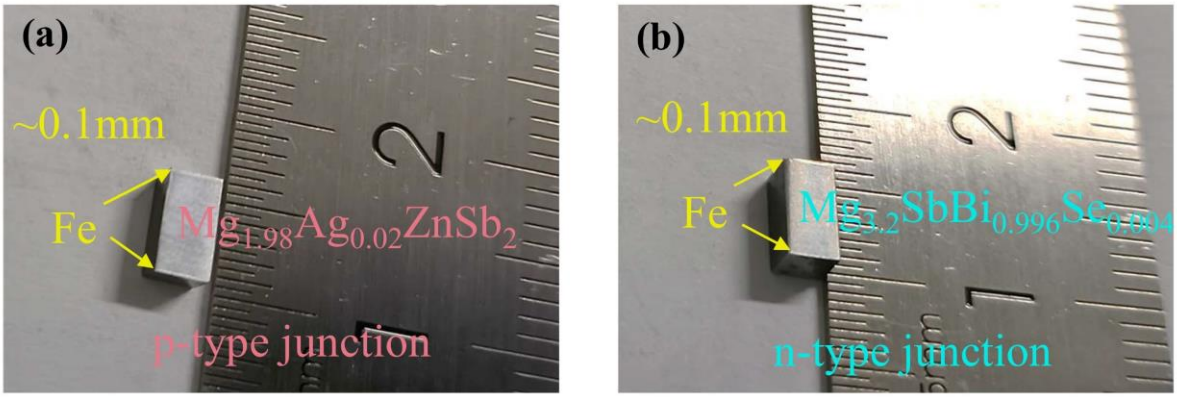

Figure S16. Photographs of **(a)** p-type and **(b)** n-type Mg_3Sb_2 legs with Fe barrier layers on two sides.

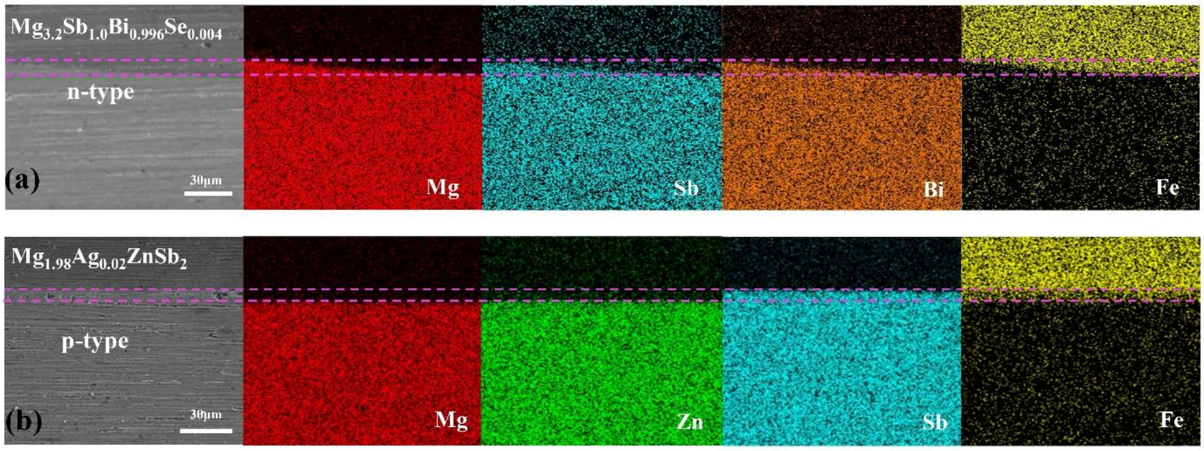

Figure S17. SEM images and the elemental mapping results of TE junctions. **(a)** n-type $Mg_{3.2}SbBi_{0.996}Se_{0.004}$/Fe and **(b)** p-type $Mg_{1.98}Ag_{0.02}ZnSb_2$/Fe.

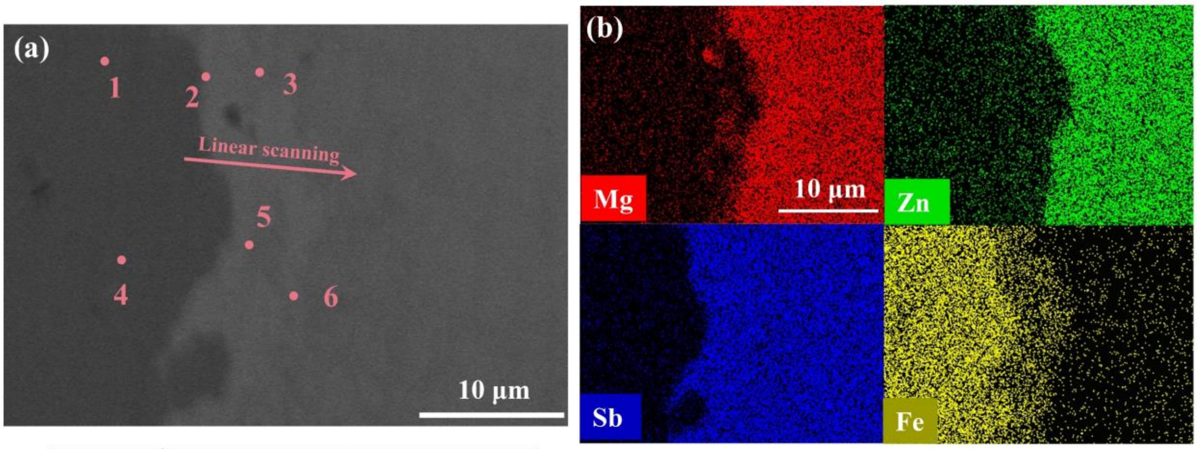

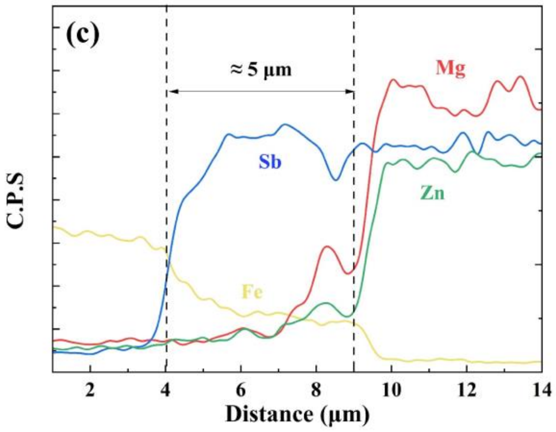

(d)

Spot	Mg (Atom, %)	Zn	Sb	Fe
1	0	0	0	100
2	1.37	0.43	39.47	58.73
3	6.38	2.77	65.27	25.58
4	0	0	0	100
5	3.11	2.42	44.18	50.30
6	42.34	16.55	41.11	0

Figure S18. SEM image and EDS results of p-type $Mg_{1.98}Ag_{0.02}ZnSb_2$/Fe interface. **(a)** SEM image. **(b)** EDS elemental mapping of Mg, Zn, Sb and Fe. **(c)** Elemental linear scanning results. **(d)** Element spot scan results.

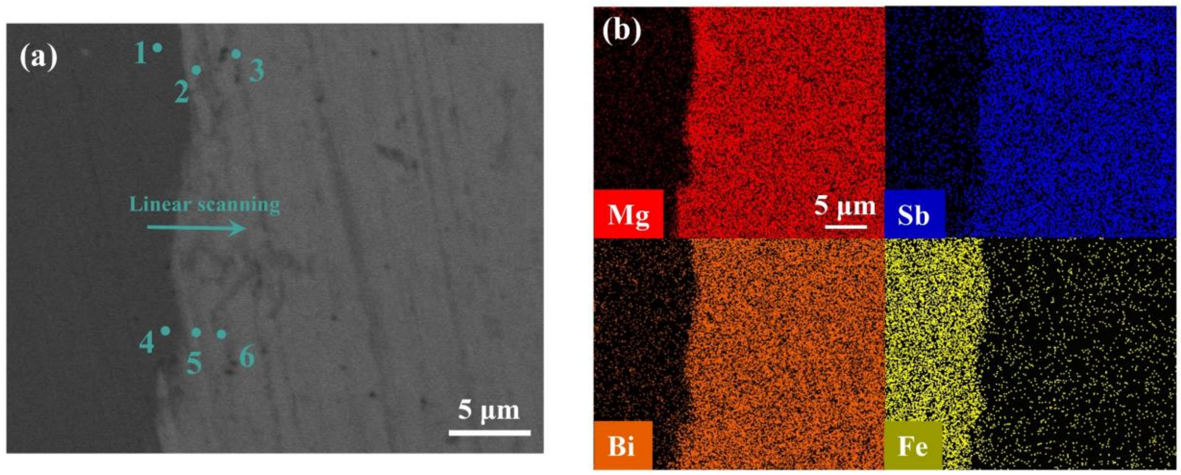

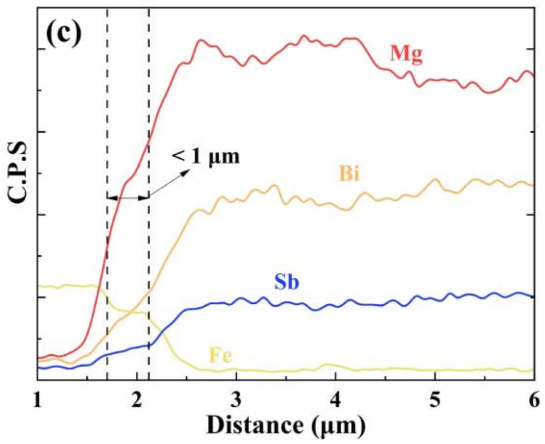

(d)

Spot	Mg (Atom, %)	Sb	Bi	Fe
1	0	0	0	100
2	41.28	11.64	9.79	37.29
3	65.98	17.16	15.46	1.40
4	0	0	0	100
5	46.64	13.94	16.23	23.19
6	60.88	21.98	17.14	0

Figure S19. SEM image and EDS results of n-type $Mg_{3.2}SbBi_{0.996}Se_{0.004}$/Fe interface. **(a)** SEM image. **(b)** EDS elemental mapping of Mg, Bi, Sb and Fe. **(c)** Elemental linear scanning results. **(d)** Element spot scan results.

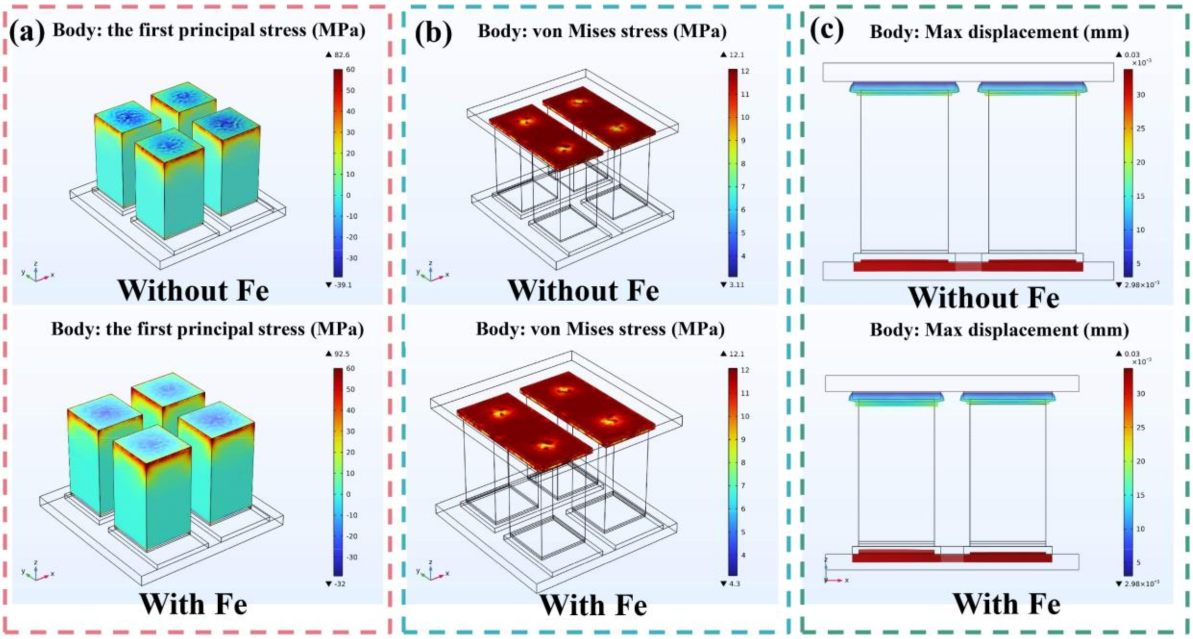

Figure S20. Cloud diagram of **(a)** distribution of simulated first principal stress in Mg_3Sb_2-based legs **(b)** simulated von Mises stress distribution in the electrodes and solders and **(c)** the maximum displacement in different components for a Mg_3Sb_2-based module before and after introducing Fe layer.

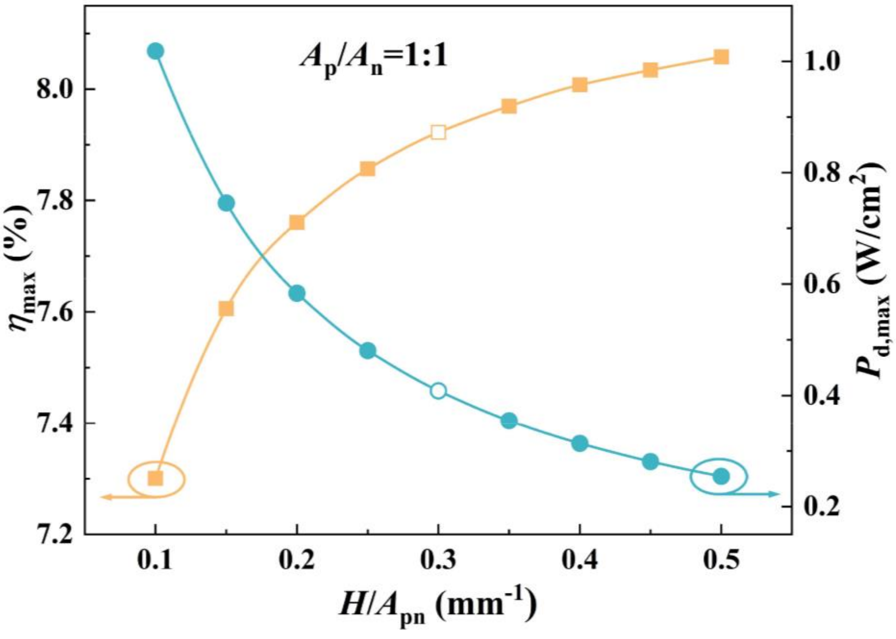

Figure S21. Simulated maximum conversion efficiency (η_{max}) and maximum power density ($P_{d,max}$) of as a function of the ratio of height to cross-sectional area (H/A_{pn}) for the fully

Mg_3Sb_2-based unicouple. Measured contact resistivities are considered in the simulations.

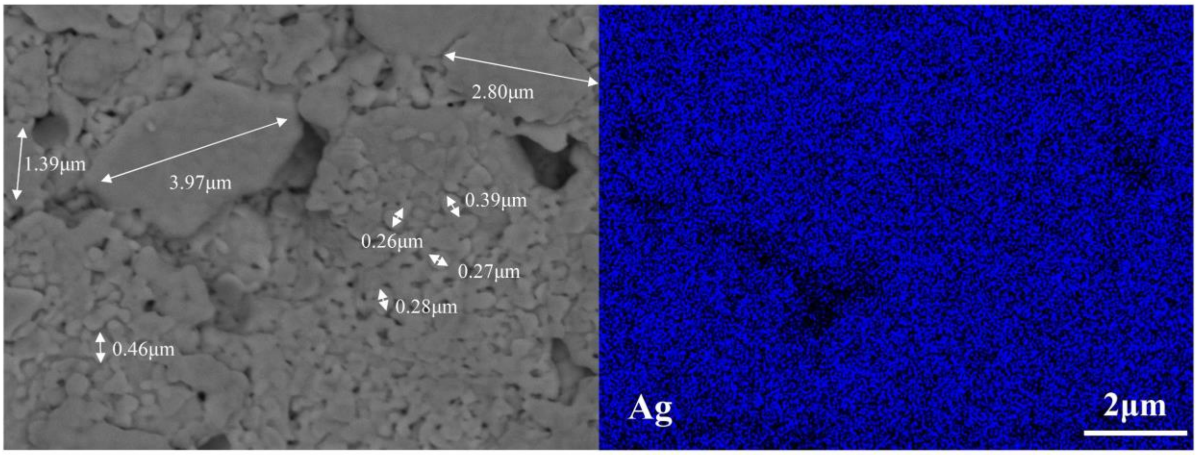

Figure S22. SEM image and the elemental mapping images for Ag composite paste consisting of nano-sized Ag particles and micron-sized Ag particles.

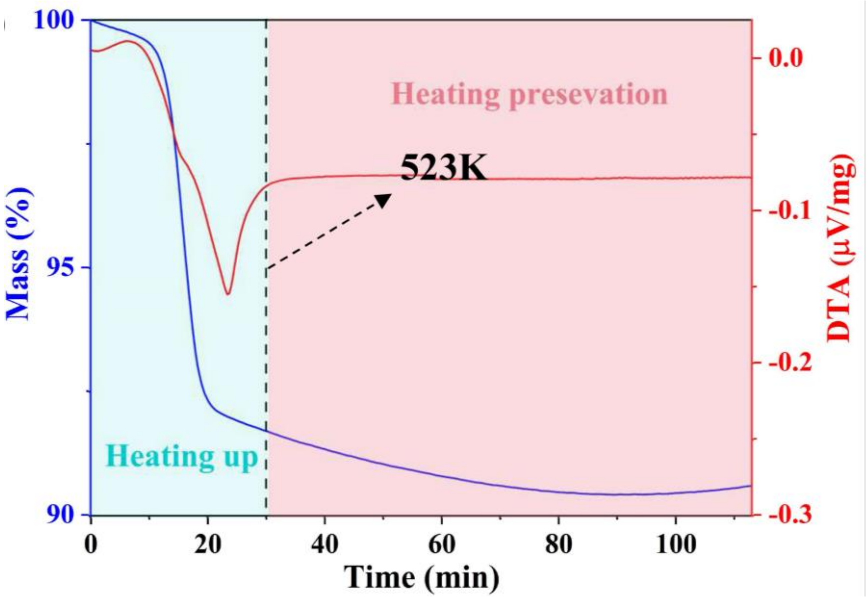

Figure S23. Mass change (TG) and heat flow (DSC) as a function of time for the Ag composite paste over heating up (from 300 K to 523 K) and heating preservation (at 523 K) processes. The Ag composite pastes can be cured at 523 K. The TG and DSC results can characterize its thermodynamic stability. We can see that the heating-up period of the first 30 minutes is the curing stage of the paste. After reaching 523 K, the holding stage adequately shows the thermodynamic stability of this paste, indicating that the minimum soldering temperature is reasonable above this temperature (523 K).

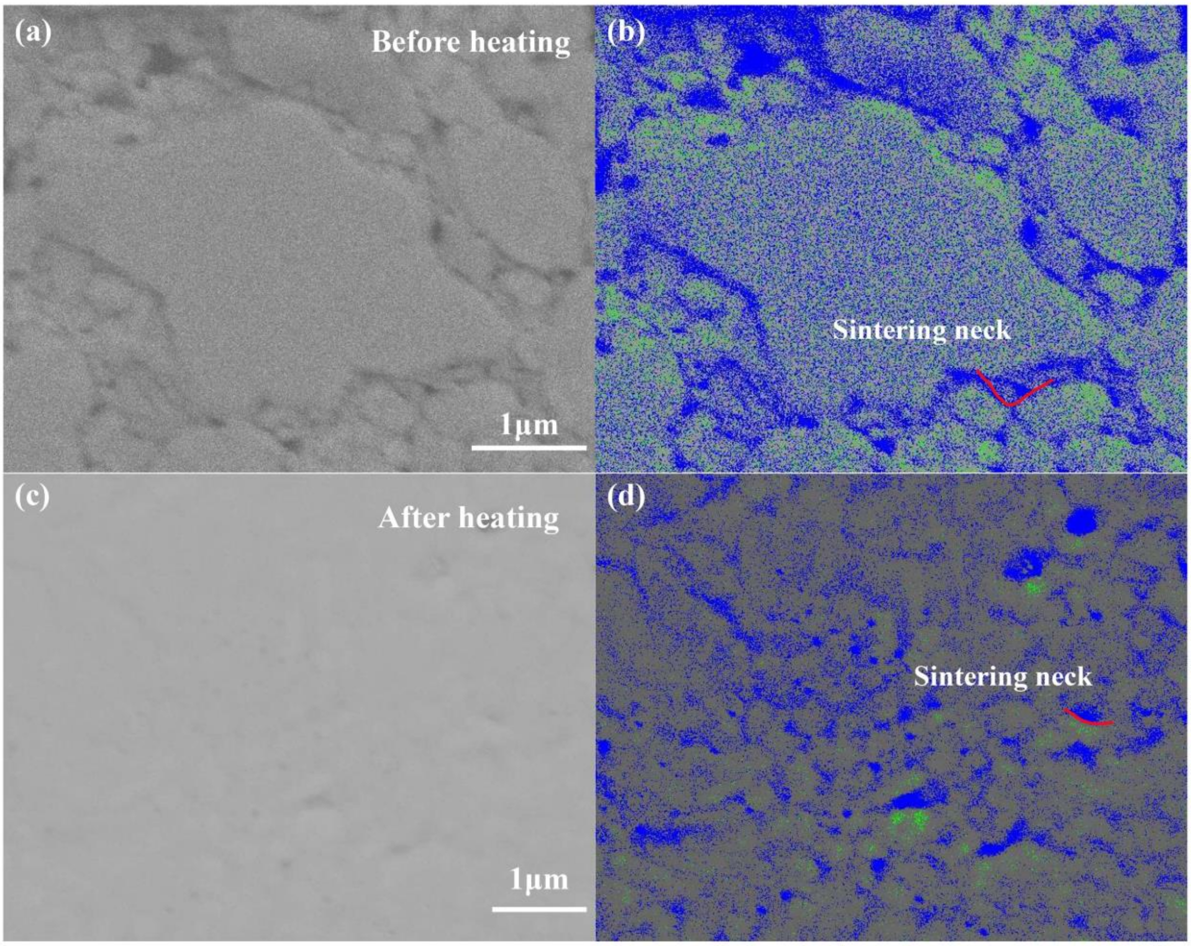

Figure S24. SEM images of the Ag composite paste (**a,b**) before heating and (**c,d**) after heating, which show the change of sintering necks.

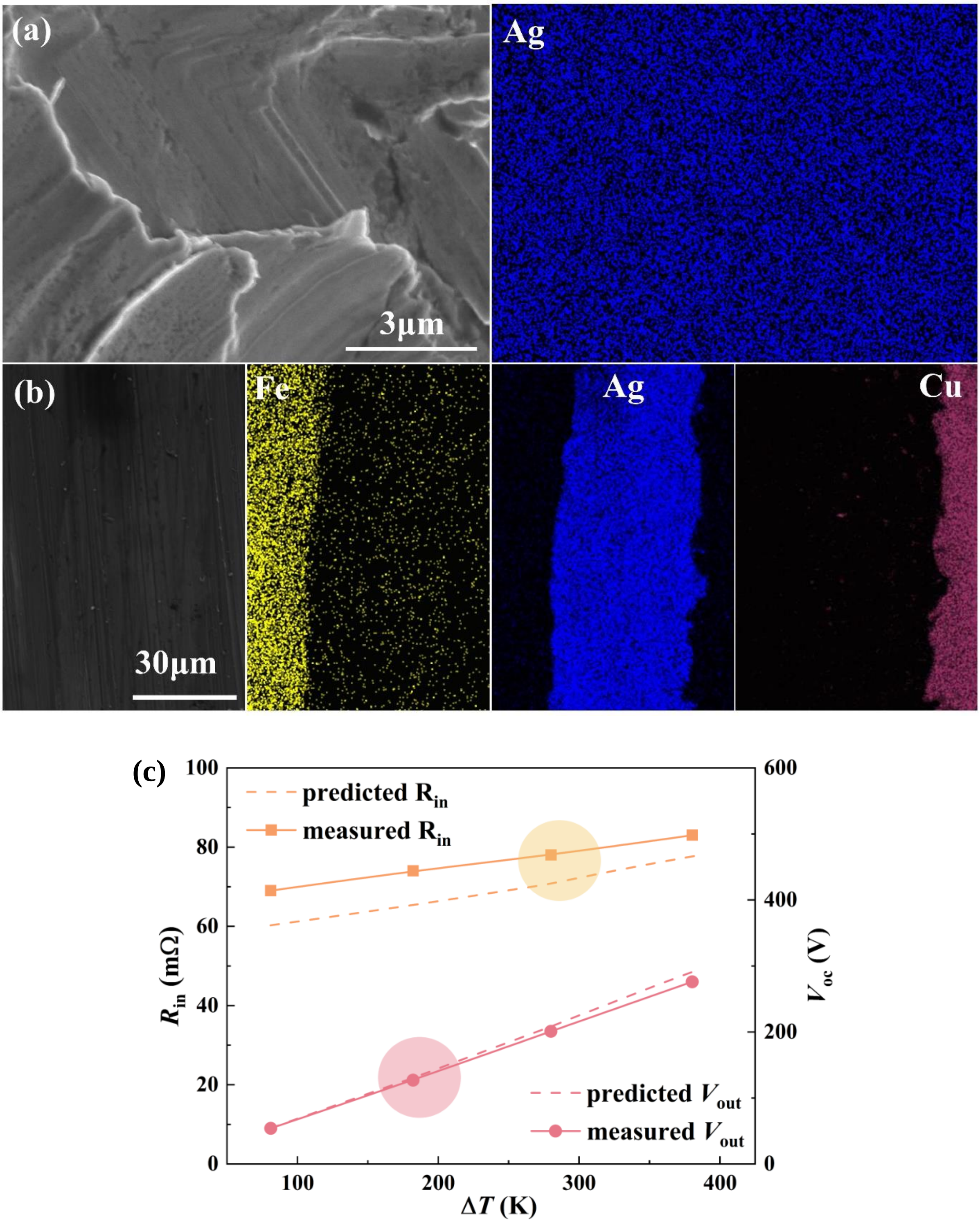

Figure S25. SEM image and the elemental mapping images for **(a)** Ag composite paste and **(b)** the joint consisting of contact layer (Fe), solder (Ag), and electrode (Cu). **(c)** Measured and predicted module resistance and open-circuit voltage as a function of temperature difference.

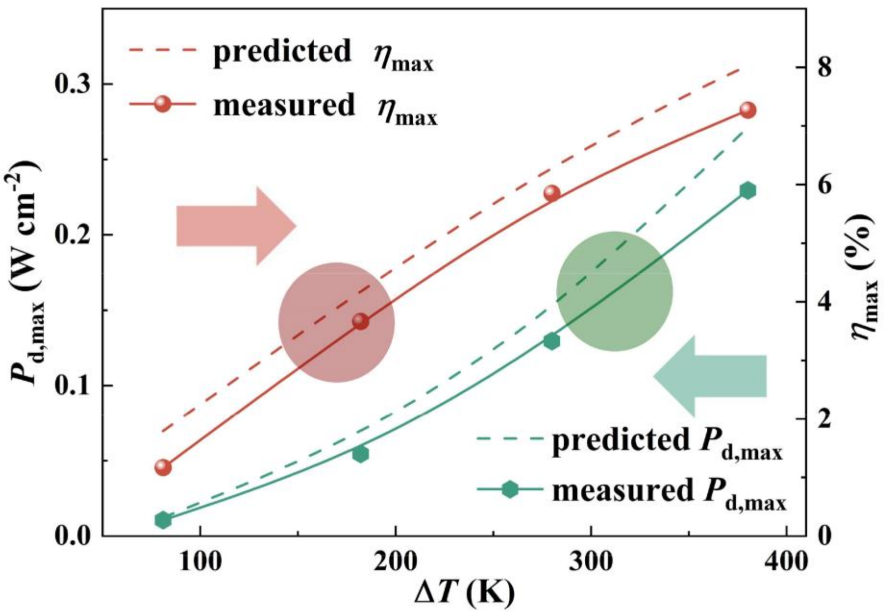

Figure S26. Measured and predicted $P_{d,max}$ (counting the occupied area of the whole module) and conversion efficiency as a function of temperature difference of our two-pair module.

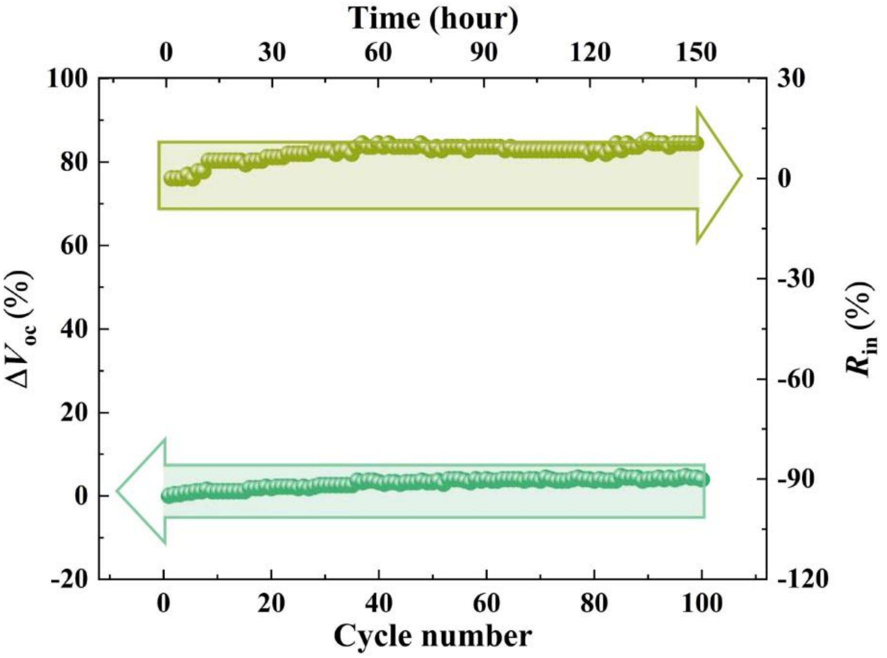

Figure S27. The change of the open circuit (ΔV_{oc}) and internal resistance (ΔR_{in}) of the Mg_3Sb_2-based same parent module during 100 thermal cycles when hot-side temperature (T_{heater}) cycles between 673 K and 473 K and cold-side temperature (T_{cooler}) is fixed at 293 K. Prior to the measurement, boron nitride coatings were sprayed onto the surfaces at hot side of the thermoelectric legs to restrain the surface oxidation and sublimation of elements. The measurement was carried out in a chamber filled with argon at an initial pressure of ~200 Pa. One cycle lasted approximately 90 min, and the module power generation performance was evaluated whenever T_{heater} was stabilized at 673 K.

The thermal cycling results show that there is a slight increase in open circuit (V_{oc}) of approximately 5%, which implies an increase in the Seebeck coefficient of the Mg_3Sb_2 materials. At the same time, the module resistance (R_{in}) also increases, by approximately 8%. The increase in R_{in} is possibly due on the one hand to an increase in resistivity of Mg_3Sb_2 because of elemental volatilization or oxidation and on the other hand to an increase in the interfacial contact resistivity.[32,33] However, taken together, the maximum output power (P_{max}) and the maximum conversion efficiency (η_{max}) remain stable and comparable to those before thermal cycling (**Figure 4d**). Given that this is the first full Mg_3Sb_2-based thermoelectric module to date, the thermal cycling results are quite encouraging. We believe that the module reliability will be substantially improved by adopting a better interfacial barrier layer (*e.g.* Mg_2Cu or Fe_7Mg_2Cr) and developing a more effective coating (*e.g.* MgO) to suppress the surface oxidation and sublimation of elements.

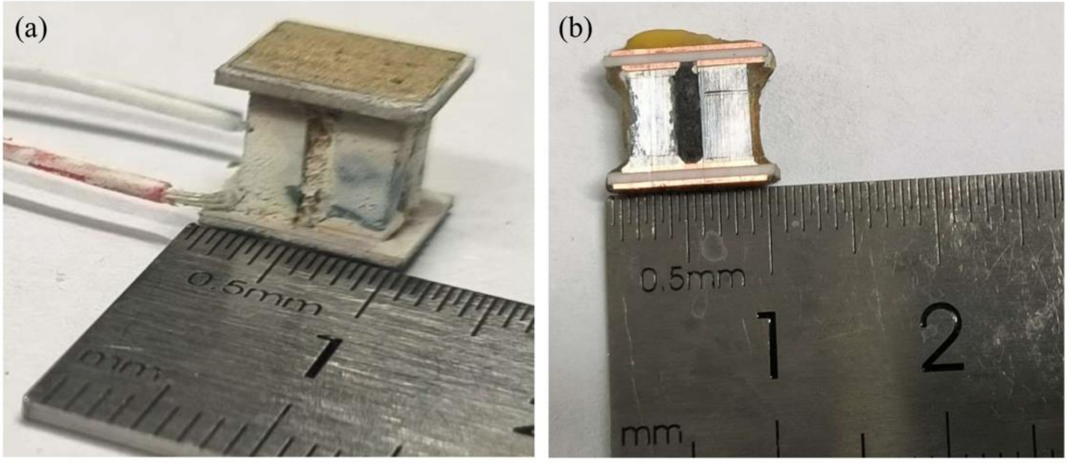

Figure S28. (**a**) Photograph of the Mg_3Sb_2 module after 100 thermal cycles, (**b**) Part of the thermocouple used for interfacial characterisation after cutting.

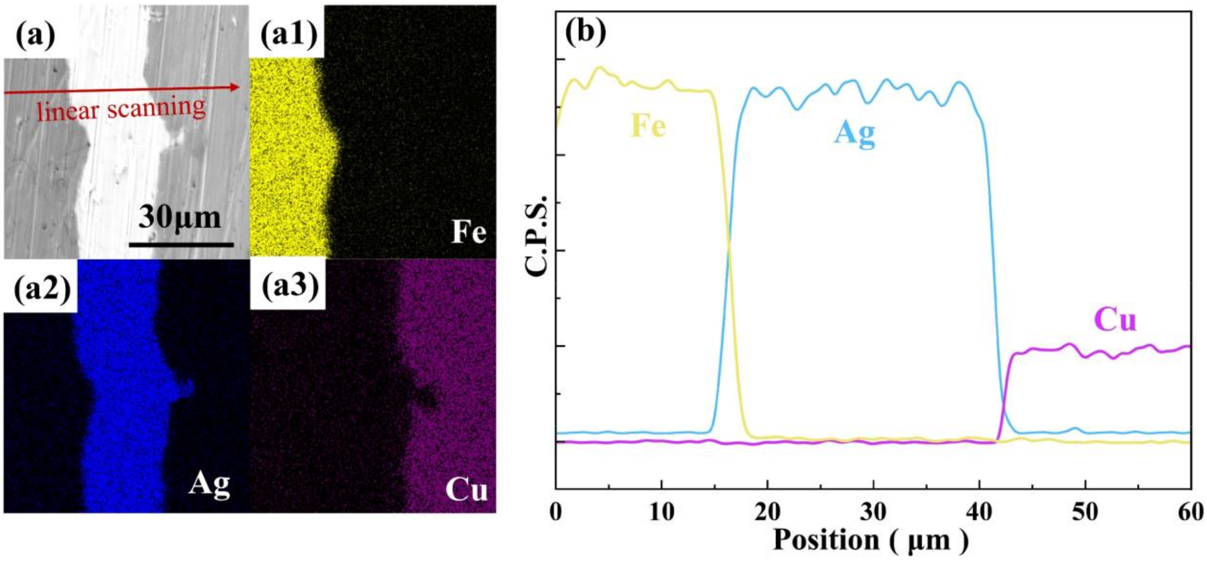

Figure S29. Microstructure of the hot-side Fe/Ag-pastes/Cu interfaces after 100 thermal cycles test. (**a**) SEM image, and EDS elemental mapping of Fe, Ag, and Cu. (**b**) Elemental linear scanning results.

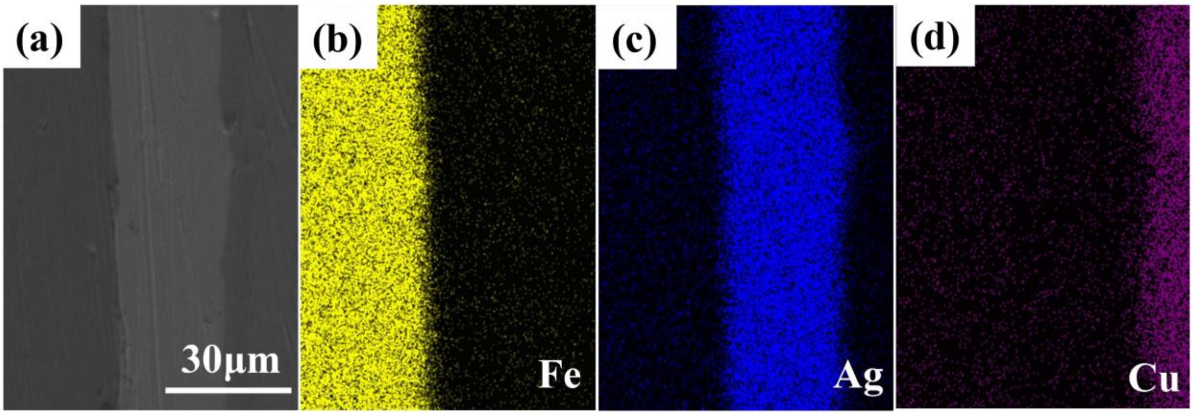

Figure S30. Microstructure of the cold-side Fe/Ag-pastes/Cu interfaces after 100 thermal cycles test. (**a**) SEM image, and EDS elemental mapping of (**b**) Fe, **c**) Ag, and **d**) Cu.

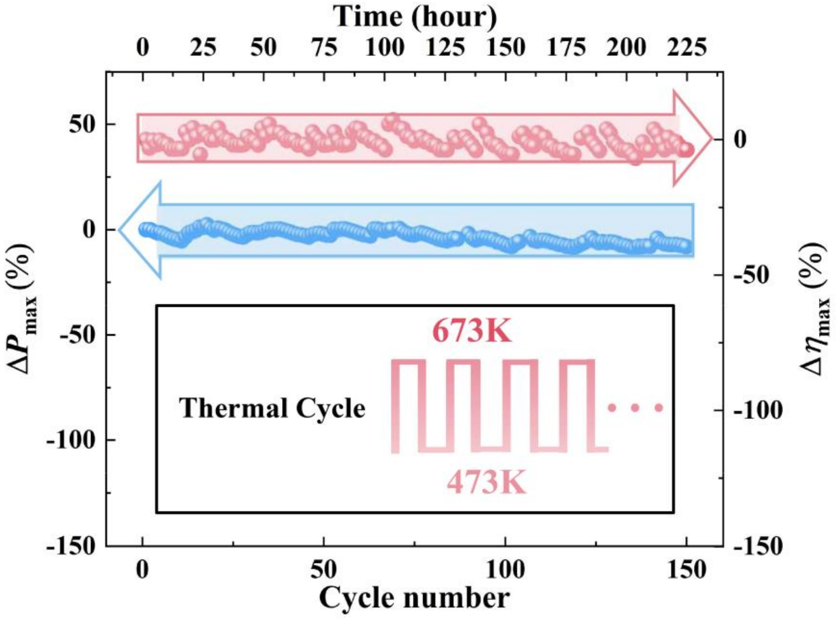

Figure S31. Change of the maximum output power (ΔP_{max}) and efficiency ($\Delta \eta_{max}$) of the 8-pair Mg_3Sb_2-based module during 150 thermal cycles when hot-side temperature (Theater) cycles between 673 K and 473 K.

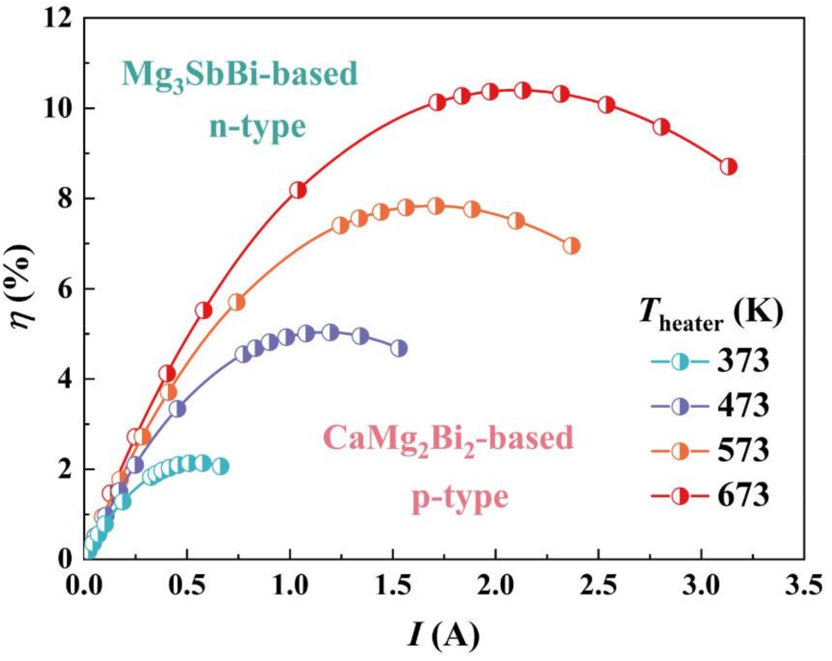

Figure S32. Predicted conversion efficiency of a module consisting of state-of-the-art n-type $Mg_{3.17}B_{0.03}Sb_{1.5}Bi_{0.49}Te_{0.01}$ and p-type $(Ca_{0.5}Yb_{0.25}Ba_{0.25})_{0.995}Na_{0.005}Mg_2Bi_{1.98}$.[34,35]

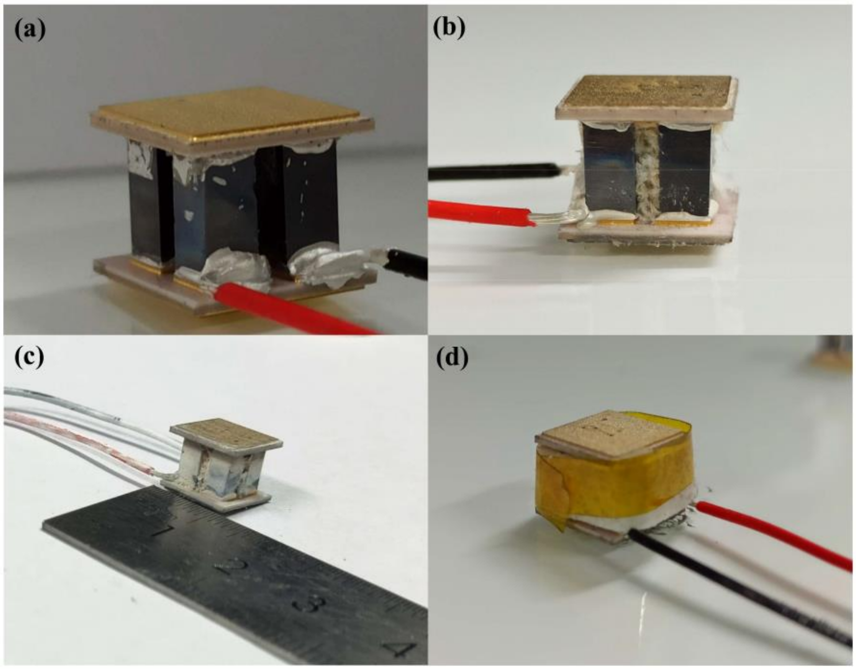

Figure S33. Photographs of the Mg_3Sb_2 module with **(a)** naked pellets, **(b)** glass fibers filled between the TE legs, **(c)** boron nitride sprayed on the p/n legs and **(d)** glass fibers wrapped around the module.

Table S1. Geometrical details of a two-pair Mg_3Sb_2-based TE module used for thermomechanical simulations.

	p/n-type legs	Ceramic substrates (Al_2O_3)	Electrodes (Cu)	Solders (Ag)
Cross section (mm^2)	3×3	10×10	2.5×4.25 / 2.5×6.5	3×3
Height/Thickness (mm)	5.5	0.63	0.3	0.1

Table S2. Results of simulated stresses using our model compared to literature[30] .

	Both constrained		2 MPa (hot side) Constrained (cold side)		Constrained (hot side) 2 MPa (cold side)	
	Max Solid. Sp1 (MPa) in					
	n-type leg	p-type leg	n-type leg	p-type leg	n-type leg	p-type leg
Our manuscript	1710	1960	149	136	304	228
Literature	1350	1950	150	117	396	311

Table S3. Examples of joining thermoelectric materials to electrodes via pressure-assisted process.

Joint components	Joining material	Joining process	Reference
$Ti_{0.3}Zr_{0.35}Hf_{0.35}CoSb_{0.8}Sn_{0.2}$/Ag/ $Ca_{2.8}Lu_{1.5}Ag_{0.05}Co_4O_{9+\delta}$/Ag	Ag foil	spark plasma sintering, at **973 K**, under **20 MPa**	[36]
$Yb_{0.25}Co_4Sb_{12}$/Mo/Ag-Cu/Mo-Cu	Ag-Cu foil	spark plasma sintering, at **1023 K,** under **10-20 MPa**	[37]
$Cu_{11}Mn_1Sb_4S_{13}$/C/Ag/Cu	Ag Paint	hot pressing, at **493 K**, under **37 MPa**	[38]
Mg_3Sb_2/Fe/AlSiCu/Cu	AlSiCu foil	hot pressing, at **830 K**, under 10 Mpa	**This work** (Elemental volatilization and cracks)
Mg_3Sb_2/Fe/Ag/Cu	Ag composite paste	tube furnace, at **523 K**, under **0.3 kPa**	**This work** (Ensure low-temperature joining and high-temperature service)

Table S4. A short summary of existing same-parent TE modules.

TE materials	Module efficiency (ΔT=380 K)	Year	Ref.
Half-Heusler	3.8%	2014	[39]
	3.9%	2015	[40]
	4.6%	2020	[41]
	4.9%	2020	[42]
	5.8%	2019	[43]
PbTe	5.0%	2018	[44]
	5.7%	2016	[45]
	6.0%	2022	[46]
$CoSb_3$	5.8%	2017	[47]
	6.2%	2017	[48]
	6.5%	2019	[49]
	6.7%	2020	[50]
Mg_3Sb_2	7.5%	2023	This work

Table S5. Summary of Mg_3Sb_2-based TE devices with emphasis on solder characteristics and device reliability evaluation.

p-type compounds	n-type compounds	Solders	Welding temperature (K)	Maximum measured temperature (K)	Device type	Device cycling evaluation conditions	Reliability results	Ref.
$Bi_{0.2}Sb_{1.8}Te_3$	$Mg_{3.2}Bi_{1.29}Sb_{0.7}Te_{0.01}$	$Pb_{97}Sn_{1.5}Ag_{1.5}$ (hot side)<br>$In_{52}Sn_{48}$ (cold side)	(Melting point ~586K)<br>(Melting point ~391K)	573	Unicouple	Cycling from 323 to 573 K, 3 cycles	Stable	[51]
$Mg_{1.594}Yb_{0.2}Na_{0.006}Zn_{1.2}Sb_2$	$Mg_{3.2}SbBi_{0.99}Te_{0.01}$	$Pb_{97}Sn_{1.5}Ag_{1.5}$ (hot side)<br>$In_{52}Sn_{48}$ (cold side)	(Melting point ~586K)<br>(Melting point ~391K)	573	Unicouple	None	None	[52]
Commercial Bi_2Te_3	$Mg_{3.2}Sb_{1.5}Bi_{0.49}Te_{0.01}Mn_{0.01}$	Sn-Ag-Cu (hot side)<br>Sn-Bi (cold side)	not mentioned	573	Unicouple	None	None	[53]
$Mg_{0.99}Cu_{0.01}Ag_{0.97}Sb_{0.99}$	$Mg_{3.2}Sb_{1.5}Bi_{0.49}Te_{0.01}Cu_{0.01}$	liquid In-Ga eutectic alloy	not mentioned	593	Module	None	None	[54]
$Cd_{0.99}Ag_{0.01}Sb$	$Mg_{3.1}Y_{0.01}SbBi$	Sn-Sb-Pb (hot side)<br>tin-based (cold side)	(Melting point ~633K)<br>(Melting point ~483K)	600	Module	Cycling from 350 to 550 K, 15 cycles, 55 hours	Stable	[55]
$(Ge_{0.98}Cu_{0.04}Te)_{0.88}(PbSe)_{0.12}$	$Mg_{3.05}Y_{0.015}SbBi$	Sn-Sb-Pb (hot side)<br>tin-based (cold side)	(Melting point ~633K)<br>(Melting point ~483K)	630	Module	None	None	[56]
$MgAg_{0.97}Sb_{0.99}$	$Mg_{3.3}Bi_{1.298}Sb_{0.7}Te_{0.002}$	Sn-based brazing material	563	548	Module	Cycling from 323 to 498 K, 10160 cycles, 1600 hours	$\triangle\eta_{max} < 5\%$	[57]
$MgAg_{0.97}Sb_{0.99}$	$Mg_{3.3}Bi_{1.498}Sb_{0.5}Te_{0.002}$<br>$Mg_{3.3}Bi_{1.298}Sb_{0.7}Te_{0.002}$	Ag paste	548	543	Module	None	None	[58]
**$Mg_{1.98}Ag_{0.02}ZnSb_2$**	**$Mg_{3.2}SbBi_{0.996}Se_{0.004}$**	**Ag composite paste**	**523**	**673**	**Module**	**Cycling from 473 to 673 K, 150 cycles, 225 hours**	**$\triangle P_{max} < 9\%$**	**This work**

							$\triangle\eta_{max} < 9\%$	

Table S6. Material properties used for simulation.[59]

Materials		d (kg cm^{-3})	α (10^{-6} K^{-1})	E (Gpa)	υ (Poisson ratio)	κ (W m^{-1} K^{-1})	C_p (J kg^{-1} K^{-1})	Yield stress (Mpa)
Ceramic substrate (Al_2O_3)		3940	7	380	0.25	30	780	/
Electrode (Cu)		8940	~17.51	~110.52	0.33	250	383~431	220-80 (300-700K)
Solder (Ag)		10490	~19	~63.5	0.38	200	235	55-32 (300-700K)
n-type leg ($Mg_{3.2}SbBi_{0.996}Se_{0.004}$)		4900	~22.7	47.52	0.32	1.0~0.87	317~342	/
p-type leg	$Mg_{1.98}Ag_{0.02}$-$ZnSb_2$	4800	~22.4	54.65	0.292	1.32~1.15	345~374	/
	$CoSb_3$	7400	~13.7	139.10	0.22	7.45~3.79	295~319	/
	GeTe	5920	~8.1	61.92	0.19	5.92~3.31	623~675	/
	FeNbSb	8460	~9.4	208.98	0.29	17.74~8.47	459~501	/
	PbTe	7860	~20.5	62.50	0.25	4.50~1.41	371~404	/
	SnSe (b-axis)	5830	~33.8	37.5	0.25	0.70~0.34	252~258	/
	α-Cu_2Se	7010	35.3	23.36	0.46	1.09~0.95	604~360	/
	β-Cu_2Se	6750	35.3	62	0.41	1.09~0.95	604~360	/
	BiCuSeO	8630	~67.4	83.2	0.30	0.86~0.54	323~353	/

REFERENCES

[1] Fu Y, Zhang Q, Hu Z *et al*. $Mg_3(Bi,Sb)_2$-based thermoelectric modules for efficient and reliable waste-heat utilization up to 750 K. *Energy Environ Sci* 2022; **15**: 3265.

[2] Xiaofang L, Qihao Z, Jincheng L *et al*. High-Efficiency Thermoelectric Power Generation Enabled by Homogeneous Incorporation of Mxene in $(Bi,Sb)_2Te_3$ Matrix. *Adv. Energy Mater*. **2019**, *10*, 1902982.

[3] Samson S, Guiqiang L, Xudong Z *et al*. Comparative study of a concentrated photovoltaic-thermoelectric system with and without flat plate heat pipe. *Energy Convers. Manag.* 2019; **193**: 1-14.

[4] Samson S, Guiqiang L, Xudong Z *et al*. Optimized high performance thermoelectric generator with combined segmented and asymmetrical legs under pulsed heat input power. *J. Power Sources* 2019; **428**: 53-66.

[5] Aminu Y and Sedat B. Modelling a Segmented Skutterudite-Based Thermoelectric Generator to Achieve Maximum Conversion Efficiency. *Appl. Sci.* 2020; **10**: 408.

[6] Alireza R. and Elias Y. Effect of substrate layers on thermo-electric performance under transient heat loads. *Energy Convers. Manag.* 2020; **219**: 113068.

[7] Simon C, Gautam D, Swatchith L *et al*. Electrodeposited Thin-Film Micro-Thermoelectric Coolers with Extreme Heat Flux Handling and Microsecond Time Response. *ACS Appl. Mater. Interfaces* 2021; **13**: 1773-82.

[8] Bhardwaj A, Chauhanab NS and Misra DK. Significantly enhanced thermoelectric figure of merit of p-type Mg_3Sb_2-based Zintl phase compounds via nanostructuring and employing high energy mechanical milling coupled with spark plasma sintering. *J. Mater. Chem. A* 2015; **3**: 10777-86.

[9] Wang Y, Zhang X, Liu Y *et al*. Significant role of nanoscale Bi-rich phase in optimizing the thermoelectric performance of Mg_3Sb_2. *Chin. Phys. B* 2019; **27**: 047212.

[10] Tang X, Zhang B, Zhang X *et al*. Enhancing the Thermoelectric Performance of p-Type Mg_3Sb_2 via Codoping of Li and Cd. *ACS Appl. Mater. Interfaces* 2020; **12**: 8359-65.

[11] Bhardwaja A and Misra DK. Enhancing thermoelectric properties of a p-type Mg_3Sb_2-based Zintl phase compound by Pb substitution in the anionic framework. *RSC Adv.* 2014; **4**: 34552-60.

[12] Chen C, Li X, Li S *et al*. Enhanced thermoelectric performance of p-type Mg_3Sb_2 by lithium doping and its tunability in an anionic framework. *J. Mater. Sci.* 2018; **53**: 16001-9.

[13] Tiadi M, Battabyal M, Jain PK *et al*. Enhancing the thermoelectric efficiency in p-type

Mg_3Sb_2 via Mg site co-doping. *Sustain. Energy Fuels* 2021; **5**: 4104-14.

[14] Hu J, Guo F, Guo M *et al*. Promoted application potential of p-type $Mg_3Sb_{1.5}Bi_{0.5}$ for the matched thermal expansion with its n-type counterpart. *J. Materiomics,* 2020; **6**: 729-35.

[15] Ren Z, Shuai J, Mao J *et al*. Significantly enhanced thermoelectric properties of p-type Mg_3Sb_2 via co-doping of Na and Zn. *Acta Mater.* 2018; **143**: 265-271.

[16] Huang L, Liu T, Mo X *et al*. Thermoelectric performance improvement of p-type Mg_3Sb_2-based materials by Zn and Ag co-doping. *Mater. Today Phys.* 2021; **21**: 100564.

[17] Niu Y, Yang C, Zhou T *et al*. Enhanced Average Thermoelectric Figure of Merit of p-Type Zintl Phase Mg_2ZnSb_2 via Zn Vacancy Tuning and Hole Doping. *ACS Appl. Mater. Interfaces* 2020; **12**: 37330-7.

[18] He Y, Day T, Zhang T *et al*. High Thermoelectric Performance in Non-Toxic Earth-Abundant Copper Sulfide. *Adv. Mater.* 2014; **26**: 3974-8.

[19] Fu C, Bai S, Liu Y *et al*. Realizing high figure of merit in heavy-band p-type half-Heusler thermoelectric materials. *Nat. Commun.* 2015; **6**: 8144.

[20] Zhu Y, Wu P, Guo J *et al*. Achieving a fine balance in mechanical properties and thermoelectric performance in commercial Bi_2Te_3 materials. *Ceramics International* 2020; **46**: 14994.

[21] Rogl G, Renk O, Ghosh S *et al*. Properties of HPT-Processed Large Bulks of p-Type Skutterudite $DD_{0.7}Fe_3CoSb_{12}$ with ZT > 1.3. *ACS Appl Energy Mater* 2021; **4**: 4831.

[22] Xing T, Song Q, Qiu P *et al*. Superior performance and high service stability for GeTe-based thermoelectric compounds. *Natl Sci Rev* 2019; **6**: 944.

[23] Silpawilawan W, Kurosaki K, Ohishi Y *et al*. FeNbSb p-type half-Heusler compound: beneficial thermomechanical properties and high-temperature stability for thermoelectrics. *J Mater Chem C* 2017; **5**: 6677.

[24] Shtern Y, Rogachev M, Bublik V *et al*. The Results of Thermal Expansion Investigation for Effective Thermoelectric Materials. *IEEE Conference of Russian Young Researchers in Electrical and Electronic Engineering*, pp. 1932–1936, *(*IEEE, EIConRus, Saint Petersburg and Moscow2019).

[25] Jin M, Tang Z, Zhang R *et al*. Growth of large size SnSe crystal via directional solidification and evaluation of its properties. *J Alloys Compd* 2020; **824**: 153869.

[26] Liu J, Li M, Yang S *et al*. Enhanced thermoelectric and mechanical properties in hierarchical tubular porous cuprous selenide. *Scr Mater* 2020; **176**: 104.

[27] Namsani S, Gahtori B, Auluck S *et al*. An interaction potential to study the thermal structure evolution of a thermoelectric material: β-Cu_2Se. *J Comput Chem* 2017; **38**:

2161.

[28] Wang J, Fu X, Zhang X *et al*. Structural, elastic, electronic, and thermodynamic properties of MgAgSb investigated by density functional theory. *Chinese Phys B* 2016; **25**: 086302.

[29] Peng W, Petretto G, Rignanese G-M *et al*. An Unlikely Route to Low Lattice Thermal Conductivity: Small Atoms in a Simple Layered Structure. *Joule* 2018; **2**: 1879-93.

[30] Li J, Huang H, Liu R *et al*. Influence of structural factors on thermal stress in skutterudite-based thermoelectric module. *Funct Mater Lett* 2021; **14**: 2151013.

[31] Viennois R, Hermet P, Beaudhuin M *et al*. Lattice Dynamics Study of Thermoelectric Oxychalcogenide BiCuChO (Ch = Se, S). *J Phys Chem C* 2019; **123**: 16046.

[32] Imasato K, Wood M, Kuo J *et al*. Improved stability and high thermoelectric performance through cation site doping in n-type La-doped $Mg_3Sb_{1.5}Bi_{0.5}$. *J Mater Chem A* 2018; **6**: 19941.

[33] Yin L, Chen C, Zhang F *et al*. Reliable N-type $Mg_{3.2}Sb1_{.5}Bi_{0.49}Te_{0.01}$/304 stainless steel junction for thermoelectric applications. *Acta Mater* 2020; **198**: 25.

[34] Chen X, Zhu J, Qin D *et al*. Excellent thermoelectric performance of boron-doped n-type Mg_3Sb_2-based materials via the manipulation of grain boundary scattering and control of Mg content. *Sci China Mater* 2021; **64**: 1761.

[35] Guo M, Zhai W, Li J *et al*. High Thermoelectric Performance of $CaMg_2Bi_2$ Enabled by Dynamic Doping and Orbital Alignment. *Adv Funct Mater* 2022; **32**: 2200407.

[36] Hung LT, Nong NV, Snyder GJ *et al*. High performance p-type segmented leg of misfit-layered cobaltite and half-Heusler alloy. *Energy Convers. Manag.* 2015; **99**: 20-7.

[37] Zhao D, Tian C, Tang S *et al*. Fabrication of a $CoSb_3$-based thermoelectric module. *Mater Sci Semicond Process* 2010; **13**: 221-4.

[38] Coelho R, Abreu YD, Carvalho F *et al*. An Electrical Contacts Study for Tetrahedrite-Based Thermoelectric Generators. *Materials* 2022; **15**: 6698.

[39] Bartholomé K, Balke B, Zuckermann D *et al*. Thermoelectric Modules Based on Half-Heusler Materials Produced in Large Quantities. *J Electron Mater* 2014; **43**: 1775.

[40] Fu C, Bai S, Liu Y *et al*. Realizing high figure of merit in heavy-band p-type half-Heusler thermoelectric materials. *Nat Commun* 2015; **6**: 8144.

[41] Xing Y, Liu R, Liao J *et al*. A Device-to-Material Strategy Guiding the “Double-High” Thermoelectric Module. *Joule* 2020; **4**: 2475.

[42] Yu J, Xing Y, Hu C *et al*. Half-Heusler Thermoelectric Module with High Conversion Efficiency and High Power Density. *Adv Energy Mater* 2020; **10**: 2000888.

[43] Xing Y, Liu R, Liao J *et al*. High-efficiency half-Heusler thermoelectric modules enabled by self-propagating synthesis and topologic structure optimization. *Energy Environ Sci* 2019; **12**: 3390.
[44] Jood P, Ohta M, Yamamoto A *et al*. Excessively Doped PbTe with Ge-Induced Nanostructures Enables High-Efficiency Thermoelectric Modules. *Joule* 2018; **2**: 1339.
[45] Hu X, Jood P, Ohta M *et al*. Power generation from nanostructured PbTe-based thermoelectrics: comprehensive development from materials to modules. *Energy Environ Sci* 2016; **9**: 517.
[46] Jia B, Huang Y, Wang Y *et al*. Realizing high thermoelectric performance in non-nanostructured n-type PbTe. *Energy Environ Sci* 2022; **15**: 1920.
[47] Zong P, Hanus R, Dylla M *et al*. Skutterudite with graphene-modified grain-boundary complexion enhances zT enabling high-efficiency thermoelectric device. *Energy Environ Sci* 2017; **10**: 183.
[48] Zhang Q, Zhou Z, Dylla M *et al*. Realizing high-performance thermoelectric power generation through grain boundary engineering of skutterudite-based nanocomposites. *Nano Energy* 2017; **41**: 501.
[49] Nie G, Li W, Guo J *et al*. High performance thermoelectric module through isotype bulk heterojunction engineering of skutterudite materials. *Nano Energy* 2019; **66**: 104193.
[50] Chu J, Huang J, Liu R *et al*. Electrode interface optimization advances conversion efficiency and stability of thermoelectric devices. *Nat Commun* 2020; **11**: 2723.
[51] Liang Z, Xu C, Shang H *et al*. High thermoelectric energy conversion efficiency of a unicouple of n-type Mg_3Bi_2 and p-type Bi_2Te_3. *Mater. Today Phys.* 2021; **19**: 100413.
[52] Liang Z, Xu C, Song S *et al*. Enhanced Thermoelectric Performance of p-Type Mg_3Sb_2 for Reliable and Low-Cost all-Mg_3Sb_2-Based Thermoelectric Low-Grade Heat Recovery. *Adv. Funct. Mater.* 2023; **33**: 2210016.
[53] Wu X, Lin Y, Han Z *et al*. Interface and Surface Engineering Realized High Efficiency of 13% and Improved Thermal Stability in $Mg_3Sb_{1.5}Bi_{0.5}$-Based Thermoelectric Generation Devices. *Adv. Energy Mater.* 2022; **12**: 2203039.
[54] Liu Z, Sato N, Gao W *et al*. Demonstration of ultrahigh thermoelectric efficiency of ∼7.3% in Mg_3Sb_2/MgAgSb module for low-temperature energy harvesting. *Joule* 2021; **5**: 1196.
[55] Bu Z, Zhang X, Hu Y *et al*. A record thermoelectric efficiency in tellurium-free modules for low-grade waste heat recovery. *Nat. Commun.* 2022; **13**: 237.
[56] Bu Z, Zhang X, Hu Y *et al*. An over 10% module efficiency obtained using non-Bi_2Te_3 thermoelectric materials for recovering heat of <600 K. *Energy Environ. Sci.* 2021; **14**:

6506.

[57] Ying P, Reith H, Nielsch K *et al*. Geometrical Optimization and Thermal-Stability Characterization of Te-Free Thermoelectric Modules Based on MgAgSb/$Mg_3(Bi,Sb)_2$. Small 2022; **18**: 2201183.

[58] Ying P, He R, Mao J *et al*. Towards tellurium-free thermoelectric modules for power generation from low-grade heat. *Nat. Commun*. 2021; **12**: 1121.

[59] Jain A, Ong S, Hautier G *et al.* Commentary: The Materials Project: A materials genome approach to accelerating materials innovation. *APL Materials* 2013; **1**: 011002.
